# Temporal changes in macrophage phenotype after peripheral nerve injury

**DOI:** 10.1186/s12974-018-1219-0

**Published:** 2018-06-15

**Authors:** Joy E. Tomlinson, Emilija Žygelytė, Jennifer K. Grenier, Michael G. Edwards, Jonathan Cheetham

**Affiliations:** 1 Cornell University College of Veterinary Medicine, Ithaca, NY USA; 2 Bioinfo Solutions LLC, Parker, CO USA

**Keywords:** Macrophage, Gene expression, Nerve injury, Regeneration

## Abstract

**Background:**

Macrophages play a key role in peripheral nerve repair and demonstrate complex phenotypes that are highly dependent on microenvironmental cues.

**Methods:**

We determined temporal changes in macrophage gene expression over time using RNA sequencing after fluorescence-activated cell sorting (FACS) macrophage populations from injured peripheral nerve. We identified key upstream regulators and dominant pathways using ingenuity pathway analysis and confirmed these changes with NanoString technology. We then investigate the effects of extreme polarizers of macrophage phenotype (IL4 and IFNγ) on nerve regeneration. We determined macrophage gene expression in vivo at the site of peripheral nerve injury with NanoString technology, and assessed recovery from sciatic nerve injury by cranial tibial muscle weights and retrograde labeling motor neurons in mice with deletion of IL4 or IFNγ receptors.

**Results:**

We demonstrate that IL4R and IFNγR deletions provide complementary responses to polarization, and alter expression of genes associated with angiogenesis and axonal extension, but do not influence recovery from peripheral nerve transection at 8 weeks after repair.

**Conclusions:**

Overall, this study provides a framework to evaluate the phenotype of macrophages over time, and provides a broader and more precise assessment of gene expression changes than has previously been commonly used. This data suggests ways in which polarization may be modulated to improve repair.

**Electronic supplementary material:**

The online version of this article (10.1186/s12974-018-1219-0) contains supplementary material, which is available to authorized users.

## Background

Macrophages play a wide range of roles during adaptive and innate immunity and can be detected in almost all tissues in the body. They are intimately involved in the processes of homeostasis, tissue repair, and regeneration [1]. The macrophage response to tissue injury is highly plastic and heavily dependent on microenvironmental cues which generate a spectrum of heterogeneous macrophage sub-populations, phenotypes, and functions. In the context of peripheral nerve injury, macrophages play a crucial role in the orchestration of series of events which are essential for successful regeneration and functional recovery to occur [2–5].

In the first 3 days after peripheral nerve injury, resident macrophages and Schwann cells (SC) remove fragmented axonal segments and breakdown the nerve–blood barrier [6]. Denervated SC then recruit hematogenous macrophages to the site of repair [7, 8]. Macrophage accumulation peaks 3 to 7 days after injury [9–12], and these infiltrating monocyte-derived cells encourage proliferation of SC [13], which fail to proliferate and transdifferentiate when migrating macrophages are excluded from the site of injury [10, 14]. Macrophages are the major cell type in the first few days after injury and secrete vascular endothelial growth factor (VEGF-A) which promotes development of the polarized microvasculature that directs SC migration within the regenerative bridge [9]. These data suggest that axonal regrowth, and so functional recovery, is heavily dependent upon the host immune response to nerve injury.

Initial classification of macrophage phenotype was based on extreme polarization states induced by in vitro ligands including interferon gamma (IFNɣ, “M1,” classical activation) [15, 16], and IL4 [15, 17–19] and IL10 (“M2,” alternative activation) [20, 21]. Extension of this system to an in vivo context has been problematic and has allowed overemphasis of a small number of markers to describe a complex spectrum of macrophage activation [15, 22]. Additionally, prior work has predominantly characterized macrophage phenotype in each experimental context using small numbers of markers [23–31]. Because in vivo macrophage phenotype is not dichotomous, conclusions drawn from studies that rely on a few markers of phenotype are likely to be highly dependent on which markers are chosen. Macrophages can have intermediate phenotypes and often show co-expression of classic M1 or M2 markers. For example, tumor-associated macrophages are often described as M2-like but they also have expression of many interferon stimulated genes [15, 32–34]. In peripheral nerve injury, a predominantly anti-inflammatory phenotype has been identified by gene expression of injured nerve stumps [35].

In this study, we characterize the temporal profile of macrophage gene expression during the early, mid, and late phases of macrophage response at the nerve injury site and identify changes in pathways associated with control of macrophage phenotype, angiogenesis, and cell migration.

We then determine differences in the global immune response to inflammation across a range of transgenic mice selected to capture extremes of macrophage polarization and identify changes in genes associated with angiogenesis and axon extension following polarization to these extremes. We then identify alterations in growth factor and neurotrophin expression associated with alterations in macrophage phenotype and evaluate the effects of macrophage polarization in vivo.

## Methods

### Animals

The following mouse strains were obtained from Jackson Laboratories: C57BL/6J (Jax # 664), BALBc/J (Jax# 651), *Il4ra*^−/−^ (BALB/c-*Il4ra*^*tm1Sz*^/J, Jax# 3514), *Ifngr1*^−/−^ (B6.129S7-*Ifngr1*^*tm1Agt*^*/*J, Jax# 3288), and *Il10rb*^−/−^ (B6.129S2-*Il10rb*^*tm1Agt*^/J, Jax# 5027). Mice with myeloid-specific deletion of Interferon gamma receptor 1 (IFNγR1) were generated by crossing mice with a conditional deletion for IFNγR1 (C57BL/6N-Ifngr1^*tm1.1Rds*^/J, Jax #015859) with myeloid-specific cre transgenic mice (Lys2cre, B6.129P2-*Lyz2*^*tm1(cre)Ifo*^/J, Jax #004781) and then backcrossing to produce mice homozygous for the floxed IFNγR1 and cre-positive in myeloid cells. The efficacy of the myeloid-specific Cre mice (Lys2cre, Jax #004781) was verified using R26r LacZ reporter mice (B6.129S4Gt(ROSA)26Sortm1Sor/J, Jax #3474). When crossed with the Lys2cre mouse, the STOP codon was excised in macrophages and lacZ was expressed. This was detected via a colorimetric assay resulting in blue coloration in the presence of β-galactosidase. LacZ localization was compared to CD68 (macrophage marker) by immunohistochemistry and expression in bone marrow-derived macrophages (BMDM), and macrophages in the intestine, dermis, and spleen was confirmed. Mice were bred in house in stable colonies, and genotypes were confirmed by PCR (Transnetyx, Cordova, TN).

Animals were allowed to acclimatize for 3 days after being brought into the research unit prior to any procedure. Rodent cages were replaced weekly. Animals were on a 12/12-h light-dark cycle and allowed food and water ad libitum. Group housing of 2–5 mice provided socialization. Daily record logs of medical procedures were maintained.

This study was performed in accordance with the Public Health Service Policy on Humane Care and Use of Laboratory Animals, the NIH guide for Care and Use of Laboratory Animals, federal and state regulations, and was approved by the Cornell University Institutional Animal Care and Use Committee.

### Sepsis model

#### LPS injection

Mice (*n* = 10–17/strain; mean age 12 weeks, range 8–18 weeks; mean weight 23 g, range 15–27 g; both sexes) were administered 50 mg/kg LPS (*Escherichia coli* O111:B4, Santa Cruz Biotechnology, Dallas, TX) diluted in 300 μl sterile saline via i.p. injection between 1 and 2 am. Animals were maintained under their normal housing conditions and were observed hourly for 48 h. Observers were blinded to mouse strain. Mice were euthanized at a humane endpoint of loss of self-righting reflex [36]. Time of death or euthanasia was recorded for each animal. Survival analysis (Gehan–Breslow–Wilcoxon) was performed using GraphPad Prism (GraphPad Software, v6).

### Bone marrow-derived macrophage (BMDM) culture

#### Culture

Culture was performed in two groups temporally: *Ifngr1*^*−/−*^, *Il10rb*^−/−^, and C57BL/6J; and *Il4ra*^−/−^ and BALB/cJ. Animals (*n* = 5/strain; mean age 6 weeks, range 5–8 weeks; female) were euthanized by CO_2_ asphyxiation, and bone marrow was harvested from the left tibia and femur. For each mouse, 5 × 10^5^ cells were seeded onto 100-mm sterile non-tissue culture-treated polystyrene petri dishes (Falcon) with 12 mL R10 cell culture medium [RPMI 1640 (Corning) with 10% FBS (ThermoFisher), 1% penicillin/streptomycin (ThermoFisher), and 1% HEPES (Sigma Aldrich)]. Cells were stimulated to differentiate into macrophages with 10 ng/mL M-CSF (eBioscience). Cells were cultured at 37°C and 5% CO_2_ for 6 days and were fed every third day. On days 6 and 7 of culture, R10+M-CSF media was refreshed for all samples. M(IFNγ+LPS) cells were stimulated with 50 ng/ml IFNγ (eBioscience) on day 6 and with 50 ng/ml IFNγ + 100 ng/ml LPS (Sigma-Aldrich) on day 7. M(IL4) macrophages were stimulated with 50 ng/ml IL-4 (BD Biosciences) on day 7. Cells were harvested on day 8.

#### Cultured macrophage harvest

Culture media was replaced with 10 ml PBS and plates incubated at 37 °C for 30 min. PBS was triturated vigorously and collected into a 50-mL conical for a total of three washes of 10 ml each. Cells were centrifuged for 10 min at 300 g and resuspended in 200 μl of 0.1% BSA in PBS. Cells were counted on a hemacytometer. 1.5 × 10^6^ cells from each sample were transferred to a v-bottom, 96-well plate (ThermoFisher Scientific) for flow cytometric labeling and analysis.

#### Flow cytometric analysis

Cultured cells were first labeled with Zombie Aqua fixable viability marker for 15 min at room temperature. Washing steps were performed with 150 μl PBS. Cells were incubated with surface markers for 45 min at 4 °C; washing steps were performed with 0.1% BSA in PBS. Dilutions were determined by titration. Cultured BMDM were labeled with anti-CD11b and CD16/32 antibodies to confirm successful differentiation to macrophages (Table 1). Cells were then fixed and permeabilized with Cytofix/Cytoperm (BD Biosciences) for 20 min at 4 °C. Once fixed, cells were incubated with antibodies to the intracellular markers arginase (Arg1) and nitric oxide synthase (Nos2) for 60 min at 4 °C (Table 1). The remaining wash steps were performed with 1× Perm/Wash (BD Biosciences) in DI water. After staining, cells were resuspended in 0.1% BSA in PBS for analysis.

**Table 1:** Antibodies used for flow cytometric analysis of cultured bone marrow-derived macrophages and for sorting macrophages from injured sciatic nerve

Antibody/probe	Clone	Fluorophore	Distributor/Cat No.	Specificity
CD16/32	2.4G2	BV-605	BD Biosciences/563006	Pan macrophage
F4/80	BM8	PE-Cy7	eBioscience/25-4801	Pan macrophage
CD11b	M1/70	Pacific Blue	BioLegend/101224	Pan macrophage
CD14	Sa2-8	PE	eBioscience/12-0141	Pan macrophage
Arg1		PE	R&D Systems/IC5868P	M2
Nos2	CXNFT	APC	eBioscience/17-5920	M1
Thy1.2	53-2.1	APC	eBioscience/17-0902	Fibroblast
CD19	ID3	APC	eBioscience/17-0193	Pan B cell
Ly6G	1A8-Ly6g	APC	eBioscience/17-9668	Granulocyte
Siglec F	ES22-10D8	APC	Miltenyi Biotec/130-102-241	Eosinophil
Ter119	Ter119	APC	eBioscience/17-5921	RBC
CD31	MEC 13.3	APC	BD Biosciences/551262	Endothelial cell
CD3e	17A2	APC	eBioscience/17-0032	T cell
Propidium iodide		na	eBioscience/00-6990	Dead cell/DNA
Zombie Aqua		na	BioLegend/423101	Fixable viability marker

*na* not available

The stained cells were analyzed by FACSAriaIII (BD Biosciences) using FACSDiva software (BD Biosciences), and final analysis was performed with FlowJo software (Tree Star, v10). The fluorochromes were excited with the instrument’s 405, 488, 532, and 633 nm lasers. The appropriate detection filters were used (Additional file 1: Table S1). Compensation beads (OneComp, eBioscience) were used to set the compensation matrix. Fluorescence was determined by gating against appropriate controls (unstained, fluorescence minus one) on samples prepared in parallel. Gates were set such that less than 1% positive events were recorded when acquiring the corresponding negative control. Cells were gated on forward and side scatter area for general cell size, forward scatter height and width to exclude doublets, and side scatter and Zombie Aqua to exclude dead cells. Cultured macrophages were defined as all viable single cells that were CD11b^+^CD16/32^+^ (Additional file 2: Figure S1b).

### Nerve injury model

#### Nerve injury

To identify changes in macrophage gene expression over time, the left sciatic nerves of BALB/cJ mice were transected proximal to the bifurcation and immediately repaired. Mice were anesthetized with 3% isoflurane and maintained under anesthesia with 2% isoflurane and oxygen. Analgesia was provided by subcutaneous meloxicam (4 mg/kg) injection pre-operatively and 24 h after surgery. The sciatic nerve was exposed and transected. Proximal and distal nerve stumps were aligned and sutured 1 mm into 5 mm nerve conduits with 10–0 suture (Ethilon) to create a non-critical defect (3 mm). For retrograde labeling of *Ifngr1* ^*fl/fl, cre/−*^ and control mice, the sciatic nerve was repaired by direct coaptation with two epineurial sutures.

#### Macrophage isolation from regenerative bridges and RNA extraction

Mice were euthanized at 3, 5, 14, or 28 days after repair to describe the early, mid, and late immune responses to nerve injury. The regenerative bridge was harvested within the conduit by transecting the proximal and distal sciatic nerve stumps 1 mm from the end of the 5 mm conduit. The epineurial sutures were cut and the regenerative bridge was removed from the conduit and placed in a petri dish with 1 mL RPMI-1640 (Corning) and was cut into 1 mm pieces. The tissues were then transferred to a 50-mL conical with 10 mL of digestion buffer. Digestion buffer comprised of 3 mg/mL collagenase type I (Sigma), 1 mg/mL hyaluronidase (Sigma), and 0.5 mL of 1 mM HEPES in RPMI-1640. After 1 h digestion in a 37 °C water bath, tissues were strained through a 70-μm mesh strainer (BD Biosciences) to obtain a single-cell suspension. Cells were centrifuged at 300 g for 10 min and resuspended in 1 mL red blood cell lysis buffer (eBioscience) for 10 min on ice then quenched with 5 mL DPBS and centrifuged again at 300 g for 10 min. The cell pellet was resuspended in 0.5% BSA (Sigma) in PBS, and cells were plated on a v-bottom 96-well plate (Nunc, Thermo Scientific) for FACS. Cells were labeled for FACS for 45 min at 4 °C using species-specific antibodies to label macrophages and other immune cells. Exclusion markers included Ly6G (granulocytes), Siglec F (eosinophils), CD19 (B lymphocytes), CD3e (T lymphocytes), Ter119 (RBC), and Thy1.2 (fibroblasts). Positive selection markers for macrophages included CD16/32, CD11b, CD14, and F4/80. Antibody details are provided in Table 1. All wash steps were performed with 0.5% BSA in PBS. Following sorting, aliquots of up to 2.0 × 10^5^ cells were placed into RNase/DNase-free 1.5-ml tubes (Eppendorf). RNA was purified according to the manufacturer’s instructions using the Quick-RNA MicroPrep kit (Zymo Research).

#### RNA sequencing

RNA sample quality was confirmed by spectrophotometry (Nanodrop) to determine concentration and chemical purity (A260/230 and A260/280 ratios) and with a Fragment Analyzer (Advanced Analytical) to determine RNA integrity. PolyA+RNA were isolated with the NEBNext Poly(A) mRNA Magnetic Isolation Module (New England Biolabs). TruSeq-barcoded RNA sequencing (RNASeq) libraries were generated with the NEBNext Ultra RNA Library Prep Kit (New England Biolabs). Each library was quantified with a Qubit 2.0 (dsDNA HS kit; Thermo Fisher) and the size distribution determined with a Fragment Analyzer (Advanced Analytical) prior to pooling. Libraries were sequenced on an Illumina NextSeq500. At least 20 M single-end 75+bp reads were generated per library. Reads were trimmed for low quality and adaptor sequences with cutadapt v1.8. parameters: -m 20 -q 20 –a AGATCGGAAGAGCACACGTCTGAACTCCAG --match-read-wildcards. Reads were mapped to the reference genome/transcriptome (Mouse: UCSC mm10) using tophat v2.0 with the following parameters: --no-novel-juncs -G <ref_genes.gtf>. Cufflinks v2.2 was used to generate FPKM values and statistical analysis of differential gene expression. In addition to statistical significance as determined by cuffdiff, the set of stringent-differentially expressed (stringent-DE) genes also had minimum twofold expression change and minimum average FPKM 5 in at least one condition. Hierarchical clustering was performed using JMP Pro 11 software (SAS Institute Inc., Cary, NC) on log2-fold change values.

#### Gene expression analysis with NanoString

Extracted RNA was incubated with a custom panel of 90 (for in vivo samples) or 114 (for in vitro samples) bar-coded probes (NanoString Technologies, Seattle, WA; Additional file 3: Table S2) specific for genes associated with macrophage phenotype and function with an emphasis on the context of peripheral nerve repair [1, 17, 37–42]. Four to five biological replicates per condition were evaluated (Molecular Biology Core Facility at Geisel School of Medicine at Dartmouth, Hanover, NH). RNA quality was evaluated with a fragment analyzer and degraded samples were replaced with an alternative sample if available. Standard NanoString protocols were followed [43, 44]. Reporter probes, hybridization solution, sample, and capture probes were mixed together and hybridized overnight at 65 °C. Following hybridization, samples were transferred and processed in the NanoString nCounter Prep Station. The Prep Station washed away excess probes and purified the target/probe complexes using magnetic beads. The purified target/probe complexes were deposited in a cartridge, laid flat, and immobilized for data collection. Data collection was carried out in the NanoString nCounter Digital Analyzer. Images were processed, and the barcodes were tabulated.

#### Effects of Il4ra and Ifngr1 deletion on macrophage gene expression and nerve regeneration

Sciatic nerve transection and repair was performed in *Il4ra*^−/−^, *Ifngr1*^*−/−*^, *Ifngr1*^fl/fl cre/−^, and control mice as described above. Macrophages were isolated from *Il4ra*^−/−^ and *Ifngr1*^*−/−*^ by FACS 3, 5 and 14 days after injury. Gene expression was determined using the custom NanoString panel. To determine the quality of repair, retrograde labeling was performed in a separate cohort of *Il4ra*^−/−^ and *Ifngr1*^fl/fl cre/−^ mice and their controls at 8 weeks after injury as previously described [45]. Briefly, mice were anesthetized and the sciatic nerve was transected 5 mm distal to the repair site. The nerve stump was soaked in 10% FluoroRuby in 2% DMSO for 1 h, rinsed, and replaced under the biceps femoris muscle. Mice were sacrificed 5 days later, perfused with chilled saline followed by 4% paraformaldehyde, and the spinal cord was harvested 5 mm cranial and 5 mm caudal to the L4-S3 region, which encompassed all retrograde labeled motor neurons. Explanted cords were fixed in 4% paraformaldehyde overnight and optically cleared with increasing concentrations of tetrahydrofuran followed by dichloromethane, and finally dibenzyl ether. [45] Spinal cords were imaged at 10× magnification with 6.4 μm z-stacks using a confocal microscope (Zeiss 510, Thornwood, NY; 561 nm). The entire motor neuron pool of all labeled cells was counted by a single-blinded observer using a customized automated cell counting program in Matlab (Release 2015a, The MathWorks, inc., Natick, MA) based on 3D Laplacian Gaussian object detection. Cranial tibial muscle weights were performed on the cohort of *Il4ra*^−/−^ and *Ifngr1*^fl/fl cre/−^ mice that underwent retrograde tracing 8 weeks after injury and repair as previously described. The cranial tibial muscles were removed prior to perfusion and weighed immediately. Cranial tibial muscle weights of the injured limb were standardized to the contralateral limb.

### Statistical analysis

#### Median fluorescent intensity (MFI)

MFI of each marker in the macrophage gate for each mouse was obtained from FlowJo (Tree star, v10). For cell culture, 4 markers (Nos2, Arg1, CD16/32, and CD11b) and 3 stimulation conditions [M(−), M(IL4), M(IFNγ+LPS)] were examined. The MFI of each marker was analyzed by linear mixed effect model on rank transformed data with mouse as a random effect and an interaction term (strain*stimulation) to determine which markers showed significant variation. Rank transformation was used due to non-homogeneity of variance and the presence of a few negative MFI values, which prevented use of log transformation. Tukey post hoc pairwise comparisons were applied.

#### Gene expression with NanoString

NanoString nSolver 2.6 Analysis Software (NanoString Technologies) was used to process raw data. Background was calculated from the raw data for each group (strain and stimulation) as the average mRNA count across all negative controls plus twice the standard deviation of the negative controls. Genes with average expression in all groups below background expression were removed from analysis. Eight potential housekeeping genes were included on the panel. Data was normalized using the housekeeping genes Actb, ANKRD27, GAPDH, TBP, and RICTOR, which were expressed in all samples and had < 60% CV. Normalized data were analyzed. Statistical analysis was performed separately by culture group, as described above, to allow for potential differences in cytokine and serum lots.

Hierarchical clustering was performed using JMP Pro 12 software (SAS Institute Inc., Cary, NC) on log transformed data to visualize gene expression patterns by strain and stimulation. ANOVA of a linear mixed effect model with mouse as a random effect and an interaction term of strain*stimulation, followed by Benjamini and Hochberg false discovery rate (FDR) correction was performed using R (R 3.2.3, RStudio Team (2015). RStudio: Integrated Development for R. RStudio, Inc., Boston, MA URL http://www.rstudio.com/) to determine which genes showed significant variation. For genes that passed FDR screening on the interaction term (FDR *p* value < 0.05), Tukey post hoc pairwise comparison was performed to examine expression differences in individual genes among the strains and stimulations. Log transformation was used to meet the assumptions of the test. Significance was set as *p* < 0.05 throughout.

#### Bioinformatics analysis

DE transcripts between time points were analyzed for over-representation of biological functions, pathways, and networks using the bioinformatics software ingenuity pathways analysis (IPA) (www.ingenuity.com) [46, 47]. IPA uses a Fisher’s exact test to identify over-represented connected biological units in a defined set of genes, which can include pathways, cellular functions, or known targets of regulatory genes. In some cases a confidence score, or *z* score, can be made on the activation state of the pathway, process, or upstream regulator based on the expression pattern of the associated genes [46]. The more positive or negative the *z* score, the more confidence the platform has that the pathway, biological process, or upstream regulator is activated or inhibited respectively.

## Results

### Temporal analysis of macrophage gene expression at the site of nerve injury in wild-type mice demonstrates complex phenotypes

To investigate the range of macrophage phenotypes expressed at the site of nerve injury, we first analyzed temporal changes in macrophage gene expression after sciatic nerve injury in wild-type mice. Macrophages were isolated from the regenerative bridge within the conduit by flow cytometry (Lin^−^F4/80^+^CD14^+^CD11b^+^CD16/32^+^, Additional file 2: Figure S1) 5, 14, and 28 days after sciatic transection and repair.

**Fig. 1:**
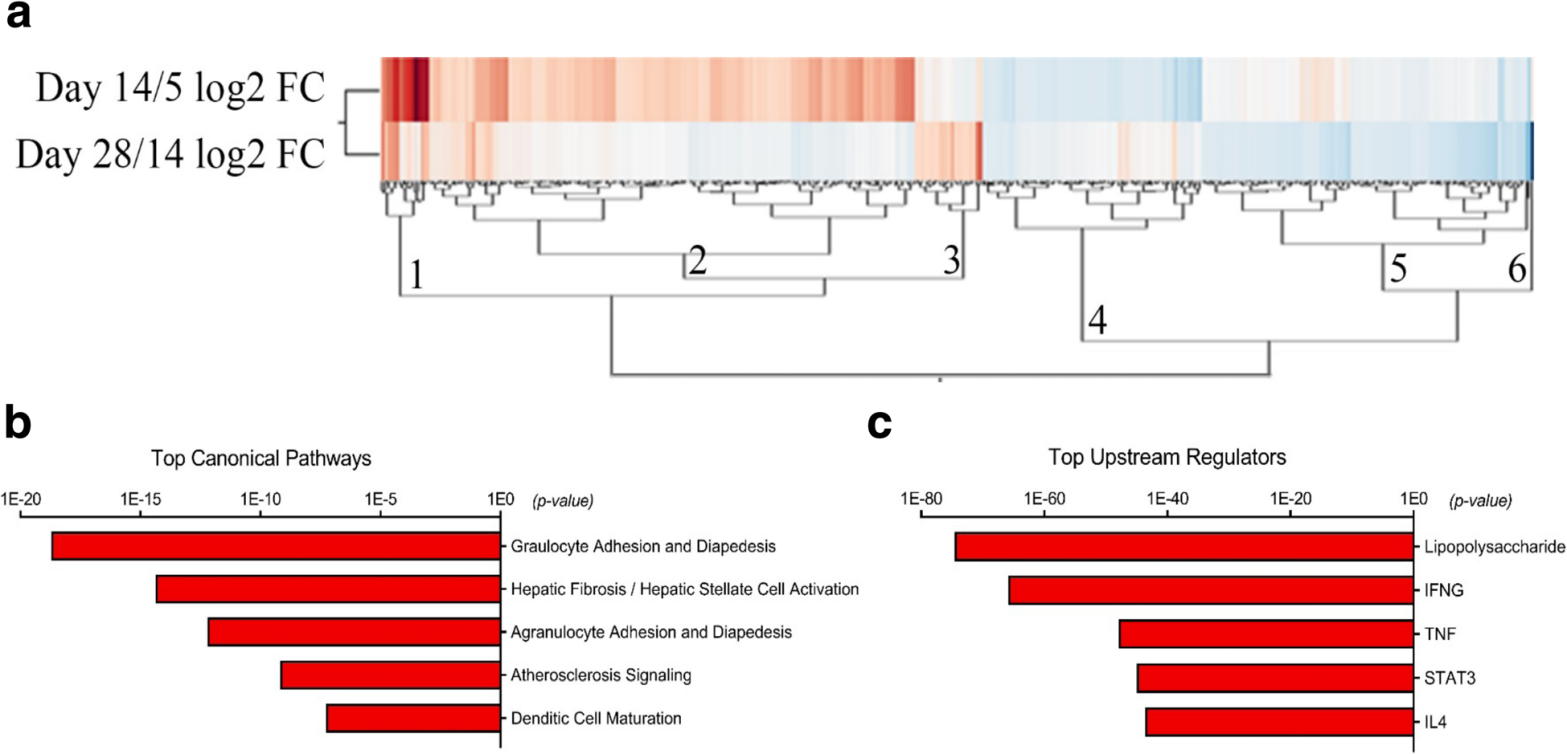
IFNɣ and IL4 are key upstream regulators of macrophage gene expression in injured nerve. **a** Unsupervised hierarchical clustering on differential gene expression over time identified six clusters of genes**.** The PANTHER GO database overrepresentation test was performed. Cluster 1 was unclassified, but contained the macrophage alternative activation marker Retnla, which increased from days 5 to 14 (red). Cluster 2 was enriched for the common biological functions of coagulation, immune response, and cell communication and included genes Alox15, Jun, and Egr1. Cluster 3 was unclassified and included genes such as Nes and Clu. Cluster 4 was dominated by cell proliferation and cytokine receptor binding, and included Chi3l3, Arg1, Ccl2, Ccl7, and Il1a, which decreased from days 5 to 14 (blue). Cluster 5 was enriched for genes related to macrophage activation and response to IFNɣ, and included Nos2. This pro-inflammatory signature declined over time. The sixth cluster contained only Sct. Red— increased with time; blue—decreased with time. **b** Ingenuity pathway analysis identified key pathways including granulocyte and agranulocyte adhesion and diapedesis, angiogenesis, and dendritic cell maturation. **c** LPS, IFNγ, STAT3, and IL4 were identified as key upstream regulators of changes in macrophage gene expression. Gene expression was compared pairwise between three time points and 382 genes met the stringent differential expression (DE) cutoff in at least one time point comparison. Stringent cutoffs of FPKM > 5 and fold change > 2 were used. RNASeq was performed on sorted macrophages from BALB/cJ mice 5, 14, and 28 days after sciatic nerve transection and repair (*n* = 3/group). Further analysis of differentially expressed (DE) transcripts between time points (days 5 vs 14; days 14 vs 28) were analyzed for over-representation of biological functions, pathways, and networks using Downstream Effects Analysis to identify those biological processes and functions that are likely to be causally affected by up- and downregulated genes, using the bioinformatics software ingenuity pathways analysis (IPA)

Gene expression was quantified with RNASeq and compared pairwise between the time points, and 382 genes met the stringent DE cutoff in at least one time point comparison. Unsupervised hierarchical clustering on differential gene expression and Panther gene ontogeny database (overrepresentation test, release 20160321) identified six clusters associated with common biological functions including macrophage activation, coagulation, immune response, cell communication, cell proliferation and cytokine receptor binding (Fig. 1a). Retnla was in cluster 1 and increased over time from day 5 to day 14, whereas other markers of alternative activation, such as Arg1 and Chi3l3, were in cluster 4 and decreased in this time period. This emphasizes the complexity of macrophage phenotype and need to include a variety of markers to accurately describe macrophage phenotype and function. DE genes between time points (d5 vs 14; d14 vs 28) were then analyzed for over-representation of biological functions, pathways, and networks using Downstream Effects Analysis through IPA to identify those biological processes and functions that are likely to be causally affected by up- and downregulated genes [46, 47]. IPA identified LPS, IFNɣ, TNF, STAT3, and IL4 as key upstream regulators of changes in macrophage polarization over time, and identified key pathways including granulocyte and agranulocyte adhesion and diapedesis, angiogenesis and dendritic cell maturation (Fig. 1b, c).

### RNASeq and NanoString data demonstrate mixed temporal expression of pro- and anti-inflammatory genes and are in agreement

We designed a custom NanoString gene expression panel, including a subset of 90 genes that were highly differentially expressed in the RNASeq data, to confirm these RNASeq findings. The NanoString platform has high reproducibility, sensitivity, and is associated with a low background signal [43, 44]. Genes were selected that describe macrophage phenotype, function, and signaling, with a preference for including pathways where multiple genes were differentially expressed in the RNASeq data. Cell lineage markers for macrophages and other cell types found in injured peripheral nerve were also included to confirm accuracy of cell sorting. Because the initial time course RNASeq data showed greater differences between days 14 and 5 than between 28 and 14, for this experiment we compared early time points of days 3, 5, and 14.

**Fig. 2:**
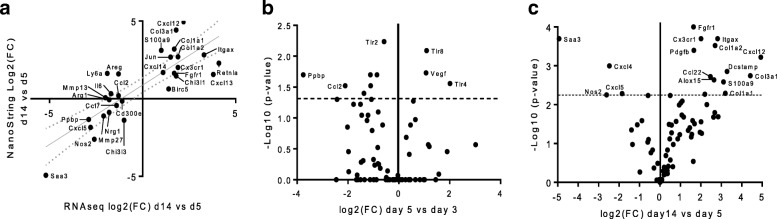
RNASeq and NanoString data demonstrate mixed expression of pro-inflammatory and anti-inflammatory genes over time. **a** Scatter plot of log2 fold changes between day 5 and 14 in macrophage gene expression detected by RNASeq (FPKM > 5) and NanoString panel. Linear regression and 95% CI are shown. **b**, **c** Differences in gene expression on NanoString panel between days 3 and 5, and 5 and 14 expressed as volcano plots. *P* values for NanoString data for 90 genes were obtained from nSolver software, with the false discovery rate (FDR) corrected level of significance (dotted line). Significance set at *p* < 0.05. Macrophages were sorted from sciatic nerves of BALB/cJ mice that were transected and repaired with an empty conduit 3, 5, or 14 days before harvest. Different mice were used for RNASeq and NanoString (*n* = 3 and 5 per time point)

NanoString data demonstrated a high level of agreement with RNASeq data (*R*^2^ = 0.67, *p* < 0.001, Fig. 2a). Greater differences were observed between macrophage gene expression at day 14 and day 5 than between day 5 and day 3 (Fig. 2b, c). Macrophage expression of the major monocyte chemokine, monocyte chemoattractant protein (CCL2), was identified early in the repair process (Fig. 2b) [48]. CX3CR1 ligand (fractalkine), which can also recruit monocytes through the CX3CR1 receptor, was identified later [49]. A variety of collagens (1a1, 1a2, and 3a1) were all elevated at day 14 compared to day 5.

While Ydens et al. [30] found no expression of the classical macrophage markers Nos2 and IL-12p40 in whole nerve lysates of the distal nerve stump up to 14 days after axotomy, we found that some IFNγ-regulated (M1) genes, such as Nos2, were initially highly expressed in macrophages isolated from the regenerative bridge and then declined from 5 to 28 days. Retnla expression increased throughout the time course, whereas Chi3l3 and Arg1 decreased from days 5 to 14 and then partially rebounded by day 28.

### Ifngr1 or Il4ra deletion profoundly alter survival after endotoxin challenge

**Fig. 3:**
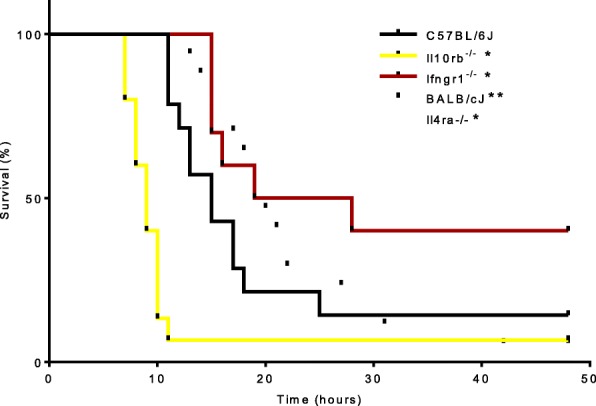
Global deletion of receptors controlling macrophage phenotype profoundly alters survival after endotoxin challenge. Gehan–Breslow–Wilcoxon survival analysis of mice administered 50 mg/kg LPS i.p. and euthanized at the humane endpoint of loss of self-righting reflex or censored at 48 h if still alive. *Ifngr1*^−/−^ mice demonstrate longer survival (*p* = 0.046) and *Il10rb*^−/−^ (*p* < 0.0001) and *Il4ra*^−/−^ (*p* = 0.014) have a shorter survival than corresponding WT. BALB/cJ survived slightly longer than C57BL/6J (*p* = 0.031). *Il10rb*^*−/−*^, and *Ifngr1*^*−/−*^ mice were on a C57BL/6J background; and *Il4ra*^*−/−*^ on a BALB/cJ background. *N* = 10–17/strain. *Significantly different survival from WT; **significantly different survival between WT strains (*p* < 0.05)

Our RNASeq/IPA data identified IFNɣ and IL4 as key regulators of macrophage phenotype in injured peripheral nerve. To further investigate this finding, we selected three mouse strains with global deletions for receptors of cytokines that control macrophage phenotype, namely *Ifngr1*^−/−^, *Il4ra*^−/−^, and *Il10rb*^−/−^, and first characterized their global inflammatory response and macrophage phenotypes. We first used a model of endotoxemia by intraperitoneal LPS injection. Mice with a less pro-inflammatory (Th1/M1) immune response are expected to have longer survival in this model. *Ifngr1* knockout reduces the ability of immune cells to respond one of the major pro-inflammatory cytokines, IFNγ, and therefore was expected to confer resistance to endotoxin. In contrast, mice with a stronger pro-inflammatory response or less ability to modulate that response should succumb more quickly. IL4 and IL10 are essential to regulate inflammation, and their knockout strains were, therefore, expected to be more susceptible to endotoxin. The strains performed as expected, with *Ifngr1*^−/−^ mice demonstrating longer survival (*p* = 0.046) and *Il10rb*^−/−^ (*p* < 0.0001) and *Il4ra*^−/−^ (*p* = 0.014) having shorter survival than their background strains (Fig. 3, Additional file 4: Table S3). Additionally, BALB/cJ are known to have a more Th2 biased immune response than C57BL/6J [15, 50–52], so these mice were expected to have better survival than C57BL/6J. The difference in immune phenotype between BALB/cJ and C57BL/6J was expected to be less than that between the three knockout strains and their controls; however, it was enough to detect a difference in survival in this model, with BALB/cJ surviving longer (*p* = 0.031), as anticipated. No differences between sexes were observed.

### Ifngr1 and Il4ra deletions provide complementary alterations in macrophage phenotype in response to in vitro polarization

The majority of macrophages involved in peripheral nerve repair are monocyte-derived (circulating) macrophages from the myeloid lineage [53, 54] and the distinct resident population is small [6] and less important [55]. To evaluate the effect of in vitro polarization on myeloid-derived macrophages from *Ifngr1*^−/−^, *Il4ra*^−/−^, and *Il10rb*^−/−^ mice, we polarized BMDM with either IFNγ+LPS or IL4. To evaluate plasticity in macrophage phenotype, we also exposed BMDM to each stimulus sequentially. We use the recently recommended nomenclature for describing in vitro macrophage polarization which is “M(stimulating ligand).” For example, cells polarized with IL4 are identified as M(IL4) instead of M2a. Polarization was confirmed using flow cytometric analysis of the commonly used macrophage phenotype markers, Nos2 (upregulated by IFNγ+LPS) and Arg1 (upregulated by IL4). As expected, expression of Nos2 and Arg1 was reduced in the absence of the receptors for a given ligand (Additional file 5: Figure S2). While CD16/32 has been frequently used as a marker of classical activation [56–59], here, we find that it was more robustly stimulated by M(IL4) than M(IFNγ+LPS) (*p* < 0.0001, Additional file 5: Figure S2). However, *Ifngr1*^−/−^ BMDM showed the most dramatic increase in CD16/32 MFI, suggesting that IFNγ signaling may modulate the effect of LPS on CD16/32 expression. The myeloid lineage marker, CD11b, showed no clear pattern of response to stimulation, with minimal differences between strains.

We then used NanoString gene expression analysis [43, 44] to expand the phenotypic description of these macrophages, because single activation markers provide an incomplete description of macrophage phenotype and because some markers can be expressed in multiple phenotypes. For example, Arg1 is expressed in some M(IFNγ+LPS) cells (Additional file 5: Figure S2a). The panel was designed to include macrophage activation markers across multiple phenotypes.

Low or absent expression of non-macrophage cell lineage markers, including Cd3e (T cells), S100b and Gfap (Schwann cells), Cd19 (B cells), and Thy1.2 (fibroblasts), confirmed the specificity of the sort. Because the panel was designed to focus on markers of activation, M(−) cells were characterized by low expression of the majority of genes in the panel. The following genes were not expressed above background in the *Ifngr1*^−/−^, *Il10rb*^−/−^, and C57BL/6J group: Alox15, Cd19, Ccl1, Ccl11, Ccl17, Ccl20, Egf, Fgf1, Fgf2, Gfap, Il5, Il10, Il23p19, Ly6g, Rtn4r, Ntf3, Ntf5, S100b, Thy1.2, Lama5, and Ngfr. The following genes were not expressed above background in the *Il4ra*^−/−^ and BALBc/J group: Alox15, Bdnf, Cd19, Ccl1, Ccl11, Ccl20, Egf, Fgf2, Gfap, Il5, Il10, Il23p19, Ly6g, Rtn4r, Ntf3, Ntf5, S100b, Thy1.2, Lama5, and Ngfr.

**Fig. 4:**
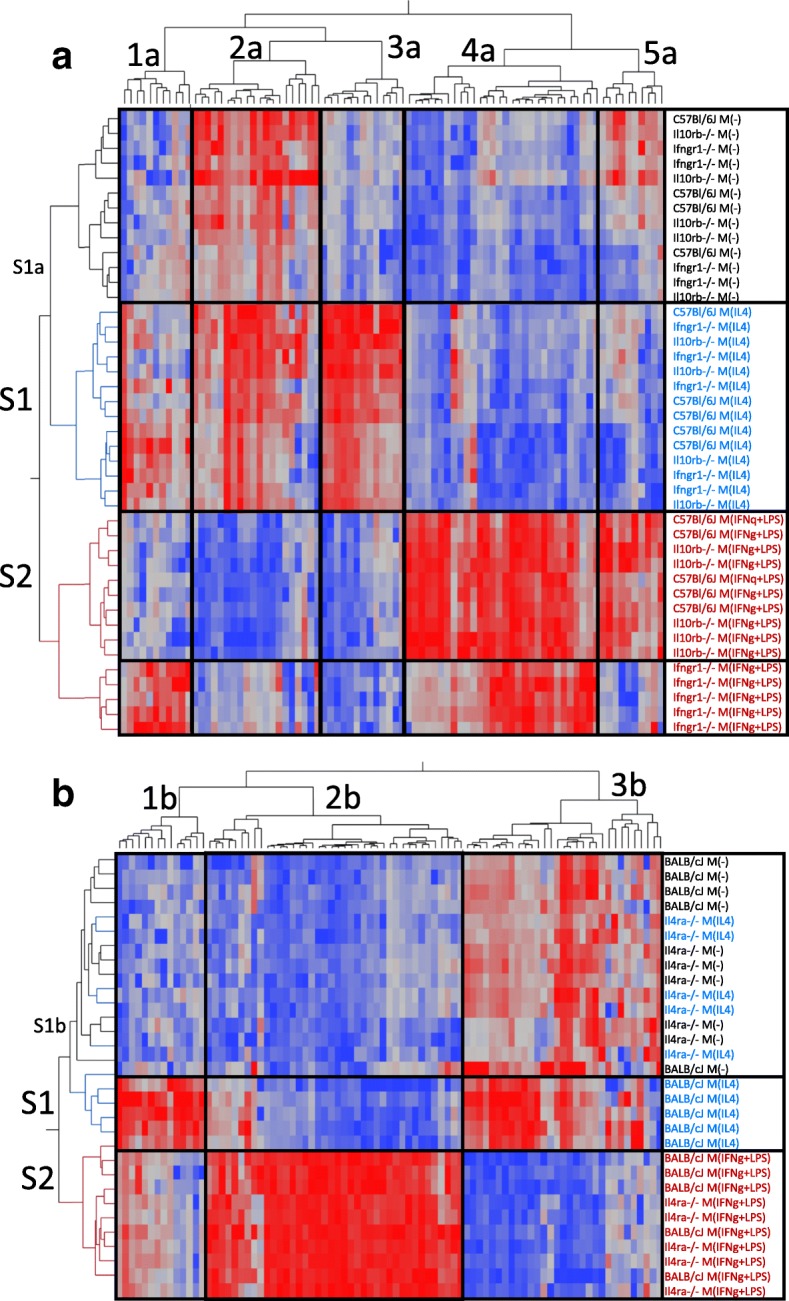
M(−) BMDM show moderate to high expression of genes that are commonly associated with a pro-regenerative/M2 phenotype. Cluster analysis of log transformed gene expression data from BMDM that were stimulated in vitro with polarizing ligands IL4 or IFNγ+LPS. Three transgenic mouse strains and their background WT strains were compared. Clustering was performed separately by background strain. In both groups, M(−) BMDM expressed many genes that are classically considered pro-regenerative or M2 (**a**, cluster 2; **b**, cluster 3). **a** *Ifngr1*^−/−^ showed a distinct pattern of gene expression after IFNγ+LPS stimulation, but *Il10rb*^−/−^ showed no difference from WT. **b** *Il4ra*^−/−^ did not respond to IL4 stimulation and *Il4ra*^−/−^ M(IL4) clustered with WT M(−). *n* = 4–5/group

Clustering analysis was performed on the NanoString data by both gene and strain-stimulation (Fig. 4). Specific genes in each cluster are reported in Additional file 6: Table S4. Clustering by strain-stimulation showed that M(−) and M(IL4) gene expression profiles tended to cluster together (row cluster S1, Fig. 4a, b), while M(IFNγ+LPS) formed a distinct cluster (row cluster S2, Fig. 4a, b). For the *Ifngr1*^*−/−*^, *Il10rb*^*−/−*^, and C57BL/6J group (Fig. 4a), column clusters 1a–3a represent genes that are highly expressed in M(IL4). Cluster 1a genes, including Cxcl13, Itgam, and Il17a*,* were also upregulated in *Ifngr1*^*−/−*^ M(IFNγ + LPS), suggesting they can be upregulated by LPS in the absence of IFNγ signaling. Cluster 2a genes were also highly expressed in M(−) macrophages and included many genes that are commonly associated with a pro-regenerative/M2 phenotype such as Cd163, Mrc1 (a.k.a CD206), and Tgfb (cluster 2a, Fig. 4a). This is consistent with prior reports that macrophages in an adherent culture with M-CSF develop a somewhat M2-like phenotype [60, 61]. Cluster 3a genes were only expressed highly in the M(IL4) group and included M2 phenotype markers such as Chil3 and Retnla. In contrast, clusters 4a and 5a represent genes that were highly expressed in M(IFNγ + LPS). Cluster 4a genes were also upregulated in *Ifngr1*^*−/−*^ M(IFNγ + LPS) compared to M(−), suggesting LPS alone was adequate to stimulate their expression to some degree. These included Stat1, Il1a, Tnf, Ccr7, Cxcl9, Il12b, Il6, and Nos2. While Ccr7, Cxcl9, Il12b, Il6, and Nos2 were upregulated in *Ifngr1*^*−/−*^ M(IFNγ + LPS) compared to M(−), it was to a significantly lesser degree than in C57BL/6J and *Il10rb*^*−/−*^ M(IFNγ + LPS). Cluster 5a genes were not upregulated in *Ifngr1*^*−/−*^ M(IFNγ + LPS), suggesting IFNγ signaling is required for their upregulation. These included Cd86, Stat3, Vegfa, and Cd80. Overall, *Il10rb*^−/−^ showed no difference from wild-type in any polarization condition (Fig. 4a), and *Ifngr1*^−/−^ mice demonstrated reduced expression of pro-inflammatory genes that are stimulated by IFNγ, such as Nos2, Ccr7, Cxcl9, Il12, and Il6, compared to WT after stimulation with IFNγ+LPS, but demonstrated minimal alteration in response to IL4 stimulation compared to WT (Fig. 4a).

For the *Il4ra*^*−/−*^ and BALB/cJ group (Fig. 4b), cluster 1b represented genes that were highly expressed in WT M(IL4) and expressed to a variable degree in M(IFNγ+LPS), which largely corresponds to cluster 3a in the *Ifngr1*^−/−^ group (Fig. 4a). Cluster 1b genes included M2 markers such as Arg1, Chil3, and Retnla. Cluster 2b (Fig. 4b) represented genes that were upregulated in M(IFNγ+LPS), corresponding to clusters 4a and 5a in the *Ifngr1*^−/−^ group (Fig. 4a). Cluster 3b represented genes that were expressed in M(−) were either up- or downregulated in WT M(IL4), and were downregulated in M(IFNγ+LPS) (Fig. 4b). These corresponded largely to cluster 2a in the *Ifngr1*^−/−^ group (Fig. 4a). *Il4ra*^−/−^ mice showed no response to IL4 stimulation and *Il4ra*^−/−^ M(IL4) clustered with WT and *Il4ra*^−/−^ M(−) in row cluster S1b (Fig. 4b). *Il4ra*^−/−^ mice had minimal to no alteration in response to IFNγ+LPS stimulation compared to WT (row cluster S2, Fig. 4b).

**Fig. 5:**
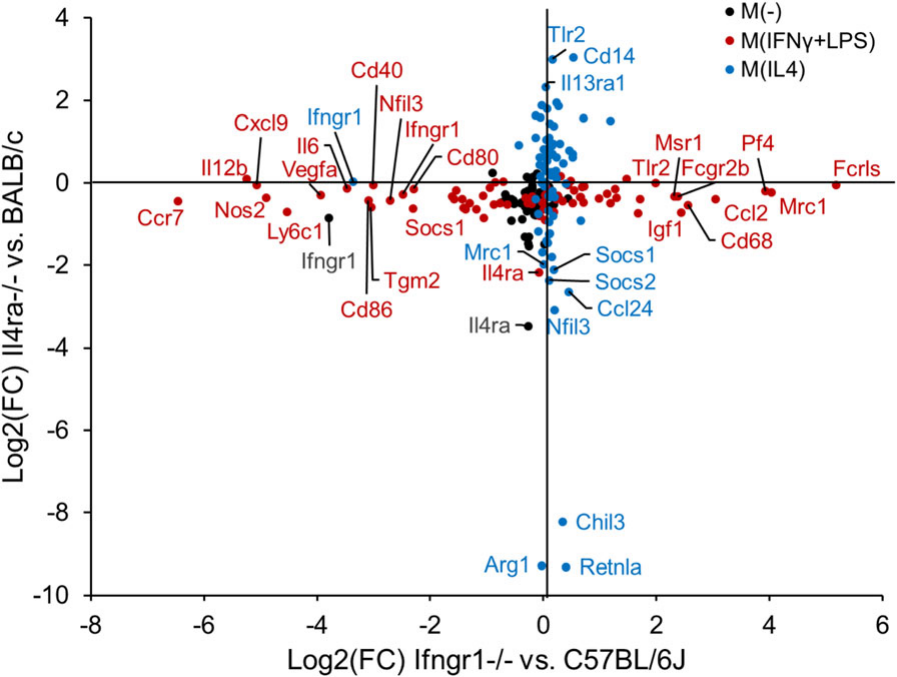
*Il4ra*^*−/−*^ and *Ifngr1*^*−/−*^ provide orthogonal alterations in macrophage phenotype in response to in vitro polarization. Gene expression Log2(fold change) over the control strain for *Il4ra*^*−/−*^ and *Ifngr1*^*−/−*^ BMDM under 3 polarization conditions: M(−), M(IL4), and M(IFNγ+LPS). *Ifngr1*^*−/−*^ macrophages demonstrate reduced expression of pro-inflammatory, or M1, markers compared to wild-type after stimulation with IFNγ+LPS, but have minimal to no alteration in response to IL4 stimulation. *Il4ra*^*−/−*^ mice have marked reduction in the classical M2 markers Arg1, Chil3, and Retnla after IL4 stimulation, with no difference in response to IFNγ+LPS stimulation. *n* = 4–5/group

In summary, mice with Ifngr1 and Il4ra deletions showed orthogonal alterations in macrophage phenotype in response to in vitro polarization (Fig. 5), thereby representing possibly useful models for studying the effects of macrophage polarization on nerve regeneration.

### Increased expression of growth factors is not limited to the M(IL4) phenotype

**Fig. 6:**
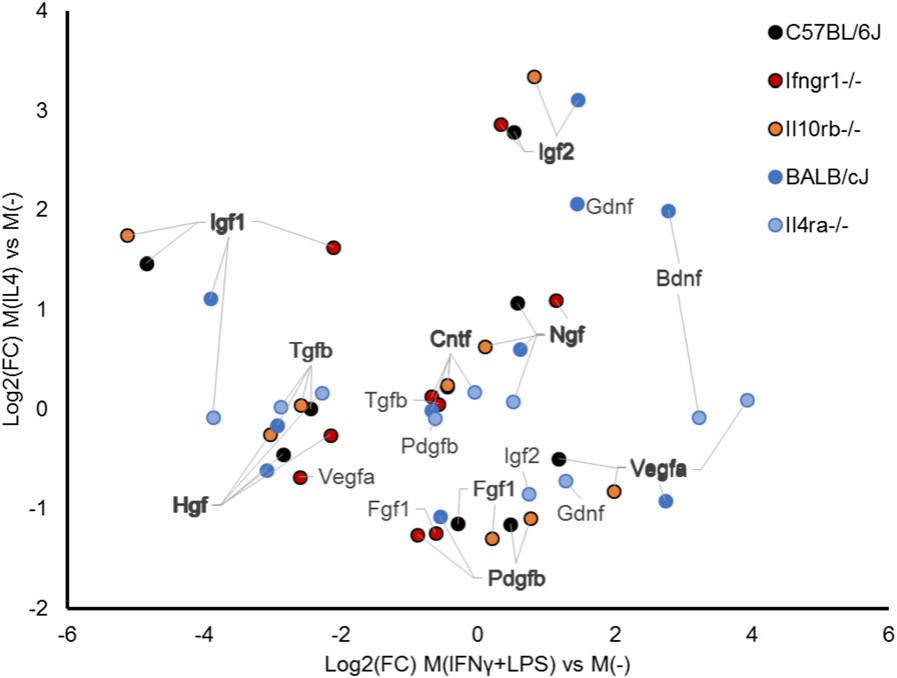
Macrophage phenotype influences gene expression of neurotrophic and growth factors relevant to peripheral nerve repair. Pro-inflammatory macrophages have higher expression of pro-angiogenic growth factors: Vegfa is consistently upregulated by IFNγ+LPS stimulation, while Pdgfb is downregulated by IL4 stimulation. Insulin-like growth factors, Igf1 and Igf2 are upregulated by IL4. Plot of gene expression Log2(fold change) over the unstimulated M(−) condition for BMDM of 5 strains of mice exposed to IL4 or IFNγ+LPS stimulation. *n* = 4–5/group

M2-like macrophages are often referred to as pro-regenerative and are associated with improved wound healing and tissue regeneration [1]. This is frequently attributed to increased production of growth factors, especially those regulating angiogenesis [1, 62–67]; however, this is controversial. There is increasing evidence that pro-inflammatory macrophages, which often predominate in early stages of tissue injury and repair [64, 65, 68, 69], are essential for the initiation of angiogenesis and attraction and proliferation of precursor cells required for repair [68–71]. We investigated expression of growth and angiogenic factors known to be important in nerve repair. Approximately half were upregulated by IL4 and half were upregulated by IFNγ+LPS (Fig. 6). We found increased Vegfa expression in M(IFNγ+LPS) which is consistent with many reports that pro-inflammatory macrophages produce more VEGF [69, 70, 72–74]. Reports surrounding PDGFb expression have been conflicting, with reports of both increased expression with alternative activation [69], and, conversely, with pro-inflammatory stimulation [75, 76]. We found PDGFb was downregulated by IL4 stimulation. Major neurotrophic factors, such as IGF1, IGF2, NGF, BDNF, NT-3, and NT-4/5 can be produced by macrophages [77–79] and are greatly upregulated in peripheral nerve injury, often concurrent with the influx of macrophages [80, 81]. We included these factors in our gene expression panel to determine whether IL4 or IFNɣ+LPS stimulation could directly induce macrophages to express neurotrophins. Igf1 was markedly downregulated by IFNɣ+LPS polarization, and Igf2 expression was markedly increased with IL4 stimulation (Fig. 6). Brain-derived neurotrophic factor (BDNF) was increased by IFNɣ+LPS stimulation; whereas hepatocyte growth factor (HGF) was reduced. NGF and CNTF expression was not affected. GDNF, NT3, and NT5 were not consistently expressed above the detection limit of the assay and, when expressed, did not show consistent response to stimulation between the control strains. It is possible that local environmental factors, such as exposure to myelin or oxidized galectin-1, are required to drive more robust expression of some neurotrophic factors [79, 82].

### Effect of Il4ra and Ifngr1 deletion on macrophage gene expression and neural regeneration after sciatic nerve transection

**Fig. 7:**
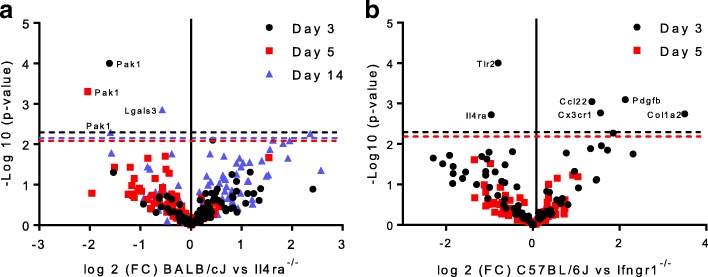
*Il4ra*^*−/−*^ and *Ifngr1*^*−/−*^ produce minimal changes in macrophage gene expression in injured nerve. **a** Macrophages from *Il4ra*^−/−^ mice had significantly higher expression of Pak1, part of the VEGF/angiogenesis pathway, at all time points (days 3, 5, and 14) compared to WT controls. **b** *Ifngr1*^*−/−*^ causes reduced expression of Col1a2, Pdgfb and Cx3cr1 (the receptor for fractaline), which are all known to be important in peripheral nerve repair. Volcano plot of NanoString data comparing the fold difference in macrophage gene expression between *Il4ra*^−/−^ mice and *Ifngr1*^*−/−*^ with WT at key time points after sciatic nerve repair. *P* values were obtained from nSolver software, with the false discovery rate corrected level of significance indicated by the horizontal dotted line. Significance set at *p* < 0.05

Based on the above data, *Ifngr1* and *Il4ra* deleted mouse strains were selected to investigate the effects of macrophage polarization on sciatic nerve regeneration. Sciatic nerves were transected and repaired as before, and macrophages were isolated from the regenerative bridge within the conduit by FACS. Macrophages from *Il4ra*^−/−^ mice had significantly higher expression of Pak1, part of the VEGF/angiogenesis pathway, at all time points (days 3, 5, and 14) compared to WT controls (Fig. 7a). Effects were more pronounced at earlier time points. Mice with global *Ifngr1*^*−/−*^ deletion had significantly lower expression of Col1a2, Cx3cr1, and Pdgfb which are all known to be important in peripheral nerve repair [66, 83, 84]. Effects were more pronounced at day 3 compared with day 5 (Fig. 7b). Overall, the effect of genotype on macrophage gene expression at the site of peripheral nerve injury in vivo was reduced compared to the effect seen in vitro.

**Fig. 8:**
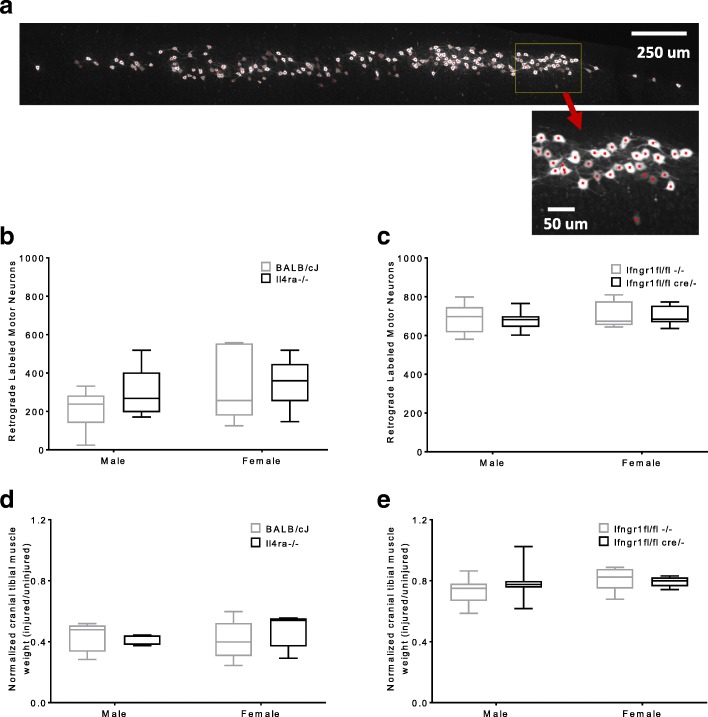
Deletion of Il4ra or Ifngr1 does not alter the quality of reinnervation. A representative image of a retrograde labeled spinal cord in shown (**a**) with red dots denoting counted cells. The total number of retrograde labeled motor neurons per mouse 8 weeks after sciatic nerve transection and repair are shown (**b**, **c**). Il4ra^−/−^ and control BALB/cJ mice underwent sciatic transection and empty conduit repair with a 3 mm gap (**b**). Ifngr1^fl/fl cre/−^ and control mice underwent sciatic transection and direct coaptation (**c**). The cranial tibial muscle weights of the injured limb normalized to the contralateral limb 8 weeks after sciatic nerve transection and repair are shown (**d**, **e**). No differences were detected between strains or sexes. *n* = 6/sex/strain, except male Ifngr1^fl/fl cre/−^ and control (*n* = 18 each). ANOVA (*p* > 0.05)

The effect of these genotypes on regeneration was examined through retrograde labeling of motor neurons, and through cranial tibial muscle weight at 8 weeks after injury. For this purpose, a myeloid-specific *Ifngr1*^*Lys2Cre fl/fl, cre/−*^ mouse had been developed and was used. No myeloid specific Il4ra knockout was available at the time and the global knockout was used. Although, some changes in macrophage gene expression had been identified with the global knockouts, myeloid-specific deletion of Ifngr1 and global deletion of Il4ra did not produce significant differences in standardized cranial tibial muscle weights or the number of retrograde-labeled motor neurons 8 weeks after injury (Fig. 8). Myeloid-specific *Ifngr1* ^*fl/fl, cre/−*^ were compared to litter-matched *Ifngr1*^*fl/fl,−/−*^ controls. No effect of sex was detected.

## Discussion

We developed an approach to isolate macrophages from peripheral nerve and characterize temporal changes in gene expression using RNA sequencing confirmed by a digital bar coded panel of 90 genes that are differentially expressed during peripheral nerve repair. We provide the first description of macrophage gene expression over time after nerve injury. RNASeq data and NanoString data showed that IL4 and IFNγ signaling pathways play pivotal roles in macrophage phenotype 5–28 days after nerve injury and that there is an early pro-inflammatory response that declines over time.

The application of causal network analysis (IPA) to RNASeq data from macrophages isolated from injured peripheral nerve allowed identification of changes in key upstream regulators of macrophage phenotype over time including IL4, IFNγ, TNF, and STAT3 [15, 17]. It also identified changes in key pathways over time including granulocyte and agranulocyte adhesion and diapedesis, angiogenesis and dendritic cell maturation. Traditionally, interpretation of high-throughput gene-expression data has been performed using statistical gene-set-enrichment methods where differentially expressed genes are intersected with sets of genes that are associated with a particular biological function or pathway [85]. A more recent approach involves the application of causal networks that integrate previously observed cause–effect relationships reported in the literature. While still depending on statistics, this is more powerful than gene-set enrichment as it leverages knowledge about the direction of effects rather than mere associations [46, 86, 87]. This causal network analysis is based on a large structured collection of observations in various experimental contexts with nearly five million findings manually curated from the biomedical literature or integrated from third-party databases. The network contains ~ 40,000 nodes that represent mammalian genes and their products, chemical compounds, microRNA molecules and biological functions. Nodes are connected by ~ 1,480,000 edges representing experimentally observed cause–effect relationships that relate to expression, transcription, activation, molecular modification, and transport as well as binding events. Network edges are also associated with a direction of the causal effect, i.e., either activating or inhibiting [46].

This study also provides a standardized description of the effects of endotoxin exposure in vivo and macrophage stimulation in vitro across a range of mouse strains commonly used to manipulate macrophage phenotype. This is the first report directly comparing the response of these mouse strains to lethal endotoxin injection. Reported survival times and lethal doses of i.p. endotoxin vary widely [88–97], preventing useful comparison between strains that are not examined concurrently. The reported variability may be partly related to endotoxin preparation and handling, as LPS rapidly binds plastics and the actual dose administered can be less than calculated. All mice used in this experiment were injected with the same batch of endotoxin, prepared simultaneously, and administered in a block randomized pattern, to minimize any variability in endotoxin concentration. This allows accurate comparison of the strains and description of their susceptibility to endotoxemia. In the LPS model of sepsis, there was markedly reduced survival in the *Il10rb*^−/−^ mice, which is consistent with findings in IL10 deficient mice [88], but which has not been previously reported.

*Ifngr1*^−/−^ and *Il4ra*^−/−^ mice provide complementary alterations in macrophage phenotype that could be useful in studying the effect of macrophages on various pathologic and regenerative processes. As expected, *Ifngr1*^−/−^ mice showed reduced polarization toward an M1-like phenotype when stimulated with IFNγ+LPS. Because this stimulation condition included LPS, the phenotype achieved in these cells was distinct from M(−) as well as from M(IFNγ+LPS) in wild-type mice. These two strains of mice provide non-overlapping alterations in macrophage phenotype that could be useful in studying the biological influence of macrophage phenotype or response to treatment with either cytokine.

In this study, we demonstrate the effect of macrophage polarization on expression of several growth factors known to be important in nerve injury, and show that pro-inflammatory as well as alternatively activated macrophages express these factors. Macrophage depletion has been shown to cause reduced angiogenesis, while exogenous addition of macrophages promotes angiogenesis [66, 98–100]. We show that pro-inflammatory stimulation of macrophages could enhance this effect because Vegfa was consistently increased by IFNɣ+LPS stimulation; while Pdgfb was consistently downregulated by IL4 stimulation. Neurotrophins are also crucial to the process of axonal regrowth and have been investigated as therapeutic targets [101–104]. M(IFNɣ+LPS) macrophages showed higher expression of Bdnf, while M(IL4) macrophages showed higher expression of Igf1, Igf2, and Hgf than M(IFNɣ+LPS) macrophages. Together these data suggest that alteration of macrophage phenotype may offer a therapeutic target mediated by altered angiogenesis or neurotrophin expression.

Despite the promising findings regarding the role of IFNɣ and IL4 signaling in peripheral nerve regeneration, we did not find a difference in quality of final regenerative outcome of mice with deletion in receptors for either cytokine. The number of motor neurons that regenerate axons across an injury site is one measure of the quality of regeneration, with greater numbers of regenerated neurons corresponding to improved fine motor control. Cranial tibial muscle weight was used as a proxy for functional recovery, with weights closer to the contralateral limb indicating greater recovery from denervation atrophy. In this study, we evaluated outcome at 8 weeks after injury, at which point mice are often maximally repaired, and we found no difference between the groups. It is possible that differences in rate of regeneration could be observed at earlier time points, or that supraphysiologic addition of exogenous cytokines could have greater effect than receptor deletion models. These are areas for future investigation.

## Conclusions

Overall, this study provides a framework to evaluate gene expression in macrophages over time at the site of peripheral nerve injury and defines the effects of specific deletions for genes that control macrophage phenotype in the context of peripheral nerve repair. This methodology provides a broader and more precise assessment of changes in macrophage gene expression at the site of nerve injury than has been previously commonly used. We also specifically assess gene expression in isolated macrophage populations rather than whole nerve lysates. Prior approaches emphasizing a small number of markers to describe a complex spectrum of macrophage activation may lead to differing conclusions dependent on which markers are chosen [15, 22]. For instance, reliance on Retnla vs. Arg1 as single markers would give opposing conclusions regarding the alternative activation status of the macrophages in these in vivo experiments. Assessing changes in macrophage gene expression in an in vivo system is important because manipulations of BMDM in vitro using ligands designed to produce extremes in polarization do not necessarily reflect changes in the context of the extracellular matrix environment and other cell types.

## Additional files

Additional file 1: **Table S1.** Excitation and detection settings for antibodies and conjugates used. (PDF 35 kb)

Additional file 2: **Figure S1.** Sorting strategy for macrophage isolation from injured nerve (a) or cell culture (b). Cells were isolated from regenerative bridges and identified as F4/80+, CD14+, CD16/32+ and CD11b+. Gates for activation markers were set by the fluorescence minus one controls such that < 1% of events were positive. (JPG 67 kb)

Additional file 3: **Table S2.** NanoString gene expression panels for in vitro and in vivo experiments (PDF 313 kb)

Additional file 4: **Table S3.** Survival after intraperitoneal endotoxin injection^1^. 1 Survival times of 5 mouse strains exposed to 50 mg/kg LPS I.P. Gehan–Breslow–Wilcoxon test and Mantel–Haenszel hazard ratios were calculated in GraphPad Prism. *Ifngr1*^*−/−*^ and *IL10rb*^*−/−*^ were compared to their background strain C57BL/6J. Il4ra^−/−^ were compared to their background strain, BALB/cJ. C57BL/6J were compared to BALB/cJ. (PDF 36 kb)

Additional file 5: **Figure S2.** In vitro stimulation of BMDM with IFNγ+LPS or IL4 resulted in differentiation into classically activated and alternatively activated macrophage phenotypes, respectively. Cross-polarization, with each stimulus delivered sequentially, resulted in intermediate phenotypes, demonstrating macrophage plasticity. Representative dot plots of cultured BMDM from (a) an *Ifngr1*^*−/−*^ mouse (red) compared to a C57BL/6J mouse (black) under M(IFNγ+LPS) stimulation condition and from (b) an*Il4ra*^*−/−*^ mouse (blue) compared to a BALB/cJ mouse (black) under M(IL4) stimulation condition with an inset histogram of the same data.Nos2 and Arg1 expression of WT strains confirm polarization to M(IFNγ+LPS) and M(IL4). (c) Median fluorescent intensity (MFI) of BMDM macrophages in the CD11b + CD16/32+ macrophage gate when exposed to 5 stimulation conditions. CD16/32 was more robustly stimulated by M(IL4) than M(IFNγ+LPS) (*p* < 0.0001). However, *Ifngr1*^−/−^ BMDM showed the most dramatic increase in CD16/32 MFI, suggesting that IFNγ signaling may modulate the effect of LPS on CD16/32 expression. CD11b showed no clear pattern of response to stimulation, with minimal differences between strains. BALB/cJ and *Il4ra*^*−/−*^ were analyzed separately, and Tukey groups are indicated by letters/numbers. Groups with the same letter or number are not significantly different. Significance was determined by linear mixed effect model with Tukey post-hoc comparison within stimulation. Significance was set as *p* < 0.05. *n* = 5 mice/strain. nd, no significant difference. (JPG 33 kb)

Additional file 6: **Table S4a.** Gene expression of bone marrow-derived macrophages from 3 mouse strains under 3 in vitro stimulation conditions. Log transformed mRNA transcript count mean +/−standard deviation & Tukey letters from linear mixed effect model with mouse as a random effect and an interaction term of strain*stimulation, followed by Benjamini and Hochberg false discovery rate (FDR) correction. *p* value for the interaction term is displayed. **Table S4b.** Gene expression of bone marrow-derived macrophages from 2 mouse strains under 3 in vitro stimulation conditions. Log transformed mRNA transcript count mean +/−standard deviation & Tukey letters from linear mixed effect model with mouse as a random effect and an interaction term of strain*stimulation, followed by Benjamini and Hochberg false discovery rate (FDR) correction. (ZIP 128 kb)

## Abbreviations

BMDM: Bone marrow-derived macrophages
DE: Differential expression
FACS: Fluorescence activated cell sorting
IPA: Ingenuity pathway analysis
MFI: Median fluorescent intensity
RNASeq: RNA sequencing
SC: Schwann cells
WT: Wild type
