# Supplementary material for: Temporal changes in macrophage phenotype after peripheral nerve injury
Source: J Neuroinflammation. 2018 Jun 15;15:185. doi: 10.1186/s12974-018-1219-0 (PMC6003127; doi:10.1186/s12974-018-1219-0)
Supplement: Supplementary file 1 — Table S1. Excitation and detection settings for antibodies and conjugates used. (PDF 35 kb) [file 12974_2018_1219_MOESM1_ESM.pdf]

Supplemental Table 1: Excitation and detection settings for antibodies and conjugates used.

Antibody target	Conjugate	Excitation laser (nm)	Emission Filters (nm)	Mirror
**CD11b**	Pacific Blue	405	450/50	
**Zombie aqua**	n/a	405	525/50	495LP
**CD16/32**	BV605	405	585/42	570LP
**Arg1, CD14**	PE	532	575/25	
**Propidium Iodide**	n/a	532	660/20	640LP
**F4/80**	PE-Cy7	532	780/40	740LP
**Nos2, Ly6G, Siglec F, CD19, CD3e, Thy1.2, Ter119, CD31**	APC	633	660/20	
