# Supplementary figures and images for: Temporal changes in macrophage phenotype after peripheral nerve injury
Source: J Neuroinflammation. 2018 Jun 15;15:185. doi: 10.1186/s12974-018-1219-0 (PMC6003127; doi:10.1186/s12974-018-1219-0)

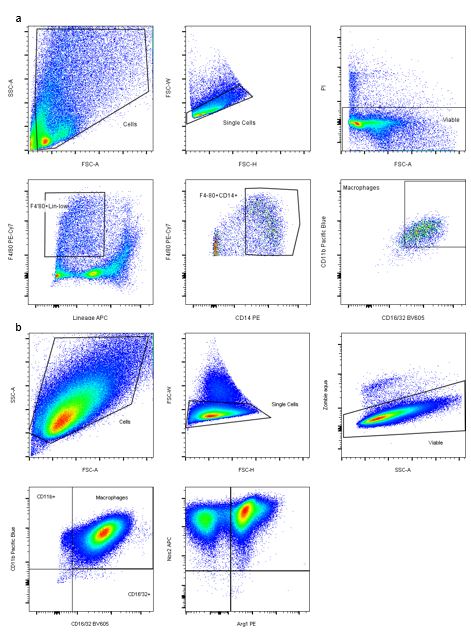

Supplement: Supplementary file 2 — Figure S1. Sorting strategy for macrophage isolation from injured nerve (a) or cell culture (b). Cells were isolated from regenerative bridges and identified as F4/80+, CD14+, CD16/32+ and CD11b+. Gates for activation markers were set by the fluorescence minus one controls such that < 1% of events were positive. (JPG 67 kb) [file 12974_2018_1219_MOESM2_ESM.jpg]

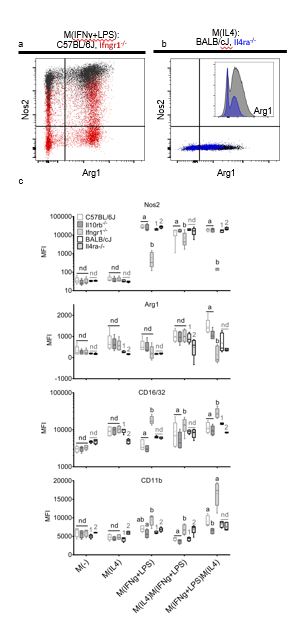

Supplement: Supplementary file 5 — Figure S2. In vitro stimulation of BMDM with IFNγ+LPS or IL4 resulted in differentiation into classically activated and alternatively activated macrophage phenotypes, respectively. Cross-polarization, with each stimulus delivered sequentially, resulted in intermediate phenotypes, demonstrating macrophage plasticity. Representative dot plots of cultured BMDM from (a) an Ifngr1−/− mouse (red) compared to a C57BL/6J mouse (black) under M(IFNγ+LPS) stimulation condition and from (b) anIl4ra−/− mouse (blue) compared to a BALB/cJ mouse (black) under M(IL4) stimulation condition with an inset histogram of the same data.Nos2 and Arg1 expression of WT strains confirm polarization to M(IFNγ+LPS) and M(IL4). (c) Median fluorescent intensity (MFI) of BMDM macrophages in the CD11b + CD16/32+ macrophage gate when exposed to 5 stimulation conditions. CD16/32 was more robustly stimulated by M(IL4) than M(IFNγ+LPS) (p < 0.0001). However, Ifngr1−/− BMDM showed the most dramatic increase in CD16/32 MFI, suggesting that IFNγ signaling may modulate the effect of LPS on CD16/32 expression. CD11b showed no clear pattern of response to stimulation, with minimal differences between strains. BALB/cJ and Il4ra−/− were analyzed separately, and Tukey groups are indicated by letters/numbers. Groups with the same letter or number are not significantly different. Significance was determined by linear mixed effect model with Tukey post-hoc comparison within stimulation. Significance was set as p < 0.05. n = 5 mice/strain. nd, no significant difference. (JPG 33 kb) [file 12974_2018_1219_MOESM5_ESM.jpg]
