# Supplementary material for: Temporal changes in macrophage phenotype after peripheral nerve injury
Source: J Neuroinflammation. 2018 Jun 15;15:185. doi: 10.1186/s12974-018-1219-0 (PMC6003127; doi:10.1186/s12974-018-1219-0)
Supplement: Supplementary file 3 — Table S2. NanoString gene expression panels for in vitro and in vivo experiments (PDF 313 kb) [file 12974_2018_1219_MOESM3_ESM.pdf]

Supplemental table 2: NanoString gene expression panels for *in vitro* and *in vivo* experiments

| | In vitro panel | In vivo panel | gene name |
|---|---|---|---|
| genes of interest | Acta2 | | smooth muscle actin |
| | Adgre1 | Adgre1 | adhesion G protein-coupled receptor Eq, a.k.a F4/80 |
| | Alox15 | Alox15 | arachidonate 15-lipoxygenase |
| | Arg1 | Arg1 | arginase 1 |
| | | Areg | amphiregulin |
| | | Birc5 | baculoviral IAP repeat containing 5 |
| | Bdnf | | brain derived neurotrophic factor |
| | | Card11 | caspase recruitment domain family member 11 |
| | Ccl1 | | C-C motif chemokine ligand 1 |
| | Ccl11 | Ccl11 | C-C motif chemokine ligand 11 |
| | Ccl17 | Ccl17 | C-C motif chemokine ligand 17 |
| | Ccl2 | Ccl2 | C-C motif chemokine ligand 2 |
| | Ccl20 | | C-C motif chemokine ligand 20 |
| | Ccl22 | Ccl22 | C-C motif chemokine ligand 22 |
| | Ccl24 | Ccl24 | C-C motif chemokine ligand 24 |
| | Ccl3 | | C-C motif chemokine ligand 3, a.k.a. MIP-1a |
| | Ccl5 | Ccl5 | C-C motif chemokine ligand 5 |
| | | Ccl7 | C-C motif chemokine ligand 7 |
| | Ccr2 | Ccr2 | C-C motif chemokine receptor 2 |
| | Ccr7 | | C-C motif chemokine receptor 7 |
| | Cd14 | Cd14 | CD14 |
| | Cd163 | | CD163 |
| | Cd19 | Cd19 | CD19 |
| | Cd3e | Cd3e | CD3e |
| | | Cd300e | CD300e |
| | Cd40 | | CD40 |
| | | Cd5l | CD5 molecule like |
| | Cd68 | | CD68 |
| | Cd80 | | CD80 |
| | Cd86 | | CD86 |
| | | Chil1 | chitinase 3-like 1 |
| | Chil3 | Chil3 | chitinase 3-like 3 |
| | Cntf | Cntf | ciliary neurotrophic factor |
| | Col6a1 | | collagen type VI alpha 1 chain |
| | | Col18a1 | collagen type XVIII alpha 1 chain |
| | | Col1a1 | collagen type I alpha 1 chain |
| | | Col1a2 | collagen type I alpha 2 chain |
| | | Col3a1 | collagen type III alpha 1 chain |
| | | Cx3cr1 | C-X3-C motif chemokine receptor 1 |
| | Cxcl1 | | C-X-C motif chemokine ligand 1. a.k.a. KC |
| | Cxcl10 | | C-X-C motif chemokine ligand 10 |
| | Cxcl11 | | C-X-C motif chemokine ligand 11 |
| | | Cxcl12 | C-X-C motif chemokine ligand 12 |
| | Cxcl13 | Cxcl13 | C-X-C motif chemokine ligand 13 |
| | | Cxcl14 | C-X-C motif chemokine ligand 14 |
| | Cxcl16 | Cxcl16 | C-X-C motif chemokine ligand 16 |

Supplemental table 2: NanoString gene expression panels for *in vitro* and *in vivo* experiments

| | In vitro panel | In vivo panel | gene name |
|---|---|---|---|
| genes of interest | | Cxcl5 | C-X-C motif chemokine ligand 5 |
| | Cxcl9 | Cxcl9 | C-X-C motif chemokine ligand 9 |
| | | Dcstamp | dendrocyte expressed seven transmembrane protein |
| | Egf | | epidermal growth factor |
| | Fcgr2b | | Fc fragment of IgG receptor II b, a.k.a. CD32 |
| | Fcgr3 | | Fc fragment of IgG receptor III, a.k.a. CD16 |
| | Fcrls | | Fc receptor-like S, a.k.a. SRAII |
| | Fgf1 | | fibroblast growth factor 1 |
| | Fgf2 | | fibroblast growth factor 2 |
| | | Fgfr1 | fibroblast growth factor receptor 1 |
| | Gdnf | Gdnf | glial cell derived neurotrophic factor |
| | Gfap | | glial fibrillary acidic protein |
| | Hgf | Hgf | hepatocyte growth factor |
| | Ifng | | interferon gamma |
| | Ifngr1 | Ifngr1 | interferon gamma receptor 1 |
| | Igf1 | Igf1 | insulin like growth factor 1 |
| | Igf2 | | insulin like growth factor 2 |
| | Il10 | Il10 | interleukin 10 |
| | Il10ra | | interleukin 10 receptor subunit alpha |
| | | Il10rb | interleukin 10 receptor subunit beta |
| | Il12b | Il12b | interleukin 12b |
| | Il13ra1 | | interleukin 13 receptor subunit alpha 1 |
| | Il17a | | interleukin 17a |
| | Il1a | Il1a | interleukin 1 alpha |
| | Il1b | Il1b | interleukin 1 beta |
| | Il1rn | | interleukin 1 receptor antagonist |
| | Il2 | | interleukin 2 |
| | Il22 | | interleukin 22 |
| | Il23a | | interleukin 23 subunit alpha |
| | Il27 | | interleukin 27 |
| | Il4ra | Il4ra | interleukin 4 receptor alpha |
| | Il5 | | interleukin 7 |
| | Il6 | Il6 | interleukin 8 |
| | Irf3 | Irf3 | interferon regulatory factor 3 |
| | Irf4 | | interferon regulatory factor 4 |
| | Irf5 | Irf5 | interferon regulatory factor 5 |
| | Itgam | Itgam | integrin subunit alpha M, a.k.a. CD11b |
| | | Itgax | integrin subunit alpha X |
| | | Jun | Jun transcription factor family |
| | Lama5 | | laminin subunit alpha 5 |
| | Lamc2 | | laminin subunit gamma 2 |
| | Lgals3 | Lgals3 | galectin 3, a.k.a. Mac-2 |
| | Lif | | LIF, interleukin 6 family cytokine, a.k.a. leukemia inhibitory factor |
| | | Ly6a | lymphocyte antigen 6 complex, locus A |

Supplemental table 2: NanoString gene expression panels for *in vitro* and *in vivo* experiments

| | In vitro panel | In vivo panel | gene name |
|---|---|---|---|
| genes of interest | Ly6c1 | | lymphocyte antigen 6 complex, locus C1 |
| | Ly6g | Ly6g | lymphocyte antigen 6 complex, locus g, a.k.a. Gr1 |
| | | Ly6i | lymphocyte antigen 6 complex, locus I |
| | Marco | | macrophage receptor with collagenous structure |
| | | Mmp12 | matrix metallopeptidase 12 |
| | | Mmp13 | matrix metallopeptidase 13 |
| | | Mmp27 | matrix metallopeptidase 27 |
| | | Myd88 | myeloid differentiation primary response 88 |
| | Mrc1 | Mrc1 | mannose receptor C-type 1, a.k.a. CD206 |
| | Msr1 | Msr1 | macrophage scavenger receptor 1, a.k.a. SRAI |
| | Nfil3 | | nuclear factor, interleukin 3 regulated |
| | Nfkbiz | Nfkbiz | NFKB inhibitor zeta |
| | Ngf | | nerve growth factor |
| | Ngfr | | nerve growth factor receptor. a.k.a. p75NTR |
| | Nos2 | Nos2 | nitric oxide synthase 2 |
| | | Nrg1 | neuregulin 1 |
| | Ntf3 | | neurotrophin 3 |
| | Ntf5 | | neurotrophin 5 |
| | | Pak1 | p21 (RAC1) activated kinase 1 |
| | Pdgfb | Pdgfb | platelet derived growth factor subunit B |
| | | Pecam1 | platelet and endothelial cell adhesion molecule 1 |
| | Pf4 | Pf4 | platelet factor 4, a.k.a. CXCL4 |
| | Pparg | | peroxisome proliferator activated receptor gamma |
| | | Ppbp | pro-platelet basic protein |
| | Retnla | Retnla | resistin like alpha, a.k.a. Fizz1 |
| | Rtn4r | | reticulon 4 receptor, a.k.a. NGR |
| | | S100a9 | S100 calcium binding protein A9 |
| | S100b | | S100 calcium binding protein B |
| | | Saa3 | serum amyloid A3 |
| | Sbno2 | | strawberry notch homolog 2 |
| | | Siglece | sialic acid binding Ig like lectin E |
| | Siglecf | | sialic acid binding Ig like lectin F |
| | Socs1 | Socs1 | suppressor of cytokine signaling 1 |
| | Socs2 | | suppressor of cytokine signaling 2 |
| | Socs3 | Socs3 | suppressor of cytokine signaling 3 |
| | Stat1 | Stat1 | signal transducer and activator of transcription 1 |
| | Stat2 | Stat2 | signal transducer and activator of transcription 2 |
| | Stat3 | Stat3 | signal transducer and activator of transcription 3 |
| | Stat6 | Stat6 | signal transducer and activator of transcription 6 |
| | Tgfb1 | Tgfb1 | transforming growth factor beta 1 |
| | Tgm2 | | transglutaminase 2 |
| | Thy1 | Thy1 | Thy1 cell surface antigen, a.k.a. CD90 |
| | Tlr1 | | toll like receptor 1 |
| | Tlr2 | Tlr2 | toll like receptor 2 |
| | Tlr4 | Tlr4 | toll like receptor 4 |

Supplemental table 2: NanoString gene expression panels for *in vitro* and *in vivo* experiments

| | In vitro panel | In vivo panel | gene name |
|---|---|---|---|
| genes of interest | Tlr8 | Tlr8 | toll like receptor 8 |
| | Tnf | Tnf | tumor necrosis factor |
| | Vegfa | Vegfa | vascular endothelial growth factor A |
| | Vim | | vimentin |
| House-keeping | Actb | Actb | actin beta |
| | Ankrd27 | Ankrd27 | ankyrin repeat domain 27 |
| | B2m | | beta 2 microglobulin |
| | Gapdh | Gapdh | glyceraldehyde-3-phosphate dehydrogenase |
| | Hmbs | | hydroxymethylbilane synthase |
| | Hprt | | hypoxanthine phosphoribosyltransferase 1 |
| | Rictor | Rictor | RPTOR independent companion of MTOR complex 2 |
| | Tbp | Tbp | TATA-box binding protein |
