# Supplementary material for: Temporal changes in macrophage phenotype after peripheral nerve injury
Source: J Neuroinflammation. 2018 Jun 15;15:185. doi: 10.1186/s12974-018-1219-0 (PMC6003127; doi:10.1186/s12974-018-1219-0)
Supplement: Supplementary file 4 — Table S3. Survival after intraperitoneal endotoxin injection1. 1 Survival times of 5 mouse strains exposed to 50 mg/kg LPS I.P. Gehan–Breslow–Wilcoxon test and Mantel–Haenszel hazard ratios were calculated in GraphPad Prism. Ifngr1−/− and IL10rb−/− were compared to their background strain C57BL/6J. Il4ra−/− were compared to their background strain, BALB/cJ. C57BL/6J were compared to BALB/cJ. (PDF 36 kb) [file 12974_2018_1219_MOESM4_ESM.pdf]

Supplemental Table 3: Survival after intraperitoneal endotoxin injection [1]

	N	Median survival (h)	Hazard ratio (95% CI of ratio)	p-value
**Ifngr1$^{-/-}$**	10	23.5	2.53 (0.941 – 6.78)	0.046
**Il10rb$^{-/-}$**	15	9	0.178 (0.0672 – 0.472)	<0.0001
**C57BL/6J**	14	15	0.630 (0.276 – 1.44)	0.031
**BALB/cJ**	17	20	-	-
**Il4ra$^{-/-}$**	14	15	0.273 (0.108 – 0.692)	0.014

[1] Survival times of 5 mouse strains exposed to 50 mg/kg LPS I.P. Gehan-Breslow-Wilcoxon test and Mantel-Haenszel hazard ratios were calculated in GraphPad Prism. *Ifngr1*$^{-/-}$ and *IL10rb*$^{-/-}$ were compared to their background strain C57BL/6J. Il4ra$^{-/-}$ were compared to their background strain, BALB/cJ. C57BL/6J were compared to BALB/cJ.
