# Supplementary material for: Temporal changes in macrophage phenotype after peripheral nerve injury
Source: J Neuroinflammation. 2018 Jun 15;15:185. doi: 10.1186/s12974-018-1219-0 (PMC6003127; doi:10.1186/s12974-018-1219-0)
Supplement: Supplementary file 6 — Table S4a. Gene expression of bone marrow-derived macrophages from 3 mouse strains under 3 in vitro stimulation conditions. Log transformed mRNA transcript count mean +/−standard deviation & Tukey letters from linear mixed effect model with mouse as a random effect and an interaction term of strain*stimulation, followed by Benjamini and Hochberg false discovery rate (FDR) correction. p value for the interaction term is displayed. Table S4b. Gene expression of bone marrow-derived macrophages from 2 mouse strains under 3 in vitro stimulation conditions. Log transformed mRNA transcript count mean +/−standard deviation & Tukey letters from linear mixed effect model with mouse as a random effect and an interaction term of strain*stimulation, followed by Benjamini and Hochberg false discovery rate (FDR) correction. (ZIP 128 kb) [file 12974_2018_1219_MOESM6_ESM.zip › Copy of Supplemental Table 4a.pdf]

Supplemental Table 4a: Gene expression of bone marrow derived macrophages from 3 mouse strains under 3 in vitro stimulation conditions. Log transformed mRNA transcript count mean +/-standard deviation & Tukey letters from linear mixed effect model with mouse a a random effect and an interaction term of strain*stimulation, followed by Benjamini and Hochberg false discovery rate (FDR) correction. p-value for the interaction term is displayed.

	Stimulation	M(-)						M(IL4)						M(IFNg+LPS)						
	Strain	C57BL/6J		Ifngr1-/-		Il10rb-/-		C57BL/6J		Ifngr1-/-		Il10rb-/-		C57BL/6J		Ifngr1-/-		Il10rb-/-		FDR
Cluster	N	4		4		5		5		5		4		5		5		5		p-value
1	Arg1	3.96 +/- 0.400	a	4.19 +/- 0.495	a	3.81 +/- 0.526	a	11.4 +/- 0.497	c	11.4 +/- 0.467	c	11.3 +/- 0.589	c	7.92 +/- 0.453	b	7.92 +/- 0.716	b	7.53 +/- 0.550	b	9.70E-01
	Ngf	3.10 +/- 0.213	ab	3.03 +/- 0.252	a	3.26 +/- 0.195	ab	3.90 +/- 0.198	d	3.76 +/- 0.314	cd	3.65 +/- 0.367	bcd	3.45 +/- 0.202	abcd	3.79 +/- 0.124	d	3.30 +/- 0.168	abc	**3.94E-02**
	Ifng	3.55 +/- 0.200	ab	3.27 +/- 0.181	a	3.46 +/- 0.256	a	3.67 +/- 0.202	ab	3.72 +/- 0.241	ab	3.72 +/- 0.298	ab	3.31 +/- 0.123	a	3.92 +/- 0.176	b	3.32 +/- 0.172	a	**3.43E-03**
	Lamc2	3.55 +/- 0.155	abc	3.47 +/- 0.188	ab	3.54 +/- 0.153	ab	3.77 +/- 0.234	bc	3.81 +/- 0.276	bc	3.79 +/- 0.137	bc	3.33 +/- 0.162	a	3.96 +/- 0.173	c	3.43 +/- 0.204	ab	**3.38E-03**
	Cd3e	3.12 +/- 0.288	ab	3.41 +/- 0.0598	ab	3.11 +/- 0.400	a	3.63 +/- 0.251	ab	3.55 +/- 0.283	ab	3.66 +/- 0.179	ab	3.27 +/- 0.183	ab	3.73 +/- 0.330	b	3.24 +/- 0.288	ab	2.36E-01
	Ccl2	7.15 +/- 1.53	a	7.27 +/- 1.13	a	6.82 +/- 1.28	a	8.18 +/- 1.57	a	8.14 +/- 1.66	a	7.94 +/- 1.92	a	8.99 +/- 0.369	ab	11.2 +/- 0.365	b	8.37 +/- 0.391	a	7.75E-02
	Cxcl13	3.65 +/- 0.205	a	3.76 +/- 0.310	a	3.61 +/- 0.160	a	3.94 +/- 0.259	ab	3.93 +/- 0.193	ab	3.94 +/- 0.181	ab	3.71 +/- 0.139	a	4.21 +/- 0.141	b	3.72 +/- 0.0801	a	5.99E-02
	Il17a	3.56 +/- 0.168	a	3.60 +/- 0.152	a	3.45 +/- 0.246	a	3.84 +/- 0.251	ab	3.74 +/- 0.291	ab	3.68 +/- 0.254	ab	3.48 +/- 0.151	a	4.06 +/- 0.0772	b	3.42 +/- 0.137	a	**4.42E-03**
	Itgam	8.16 +/- 0.0619	cd	8.15 +/- 0.0774	cd	8.16 +/- 0.0846	d	7.73 +/- 0.185	bc	7.75 +/- 0.0992	bcd	7.67 +/- 0.160	b	7.46 +/- 0.264	b	8.74 +/- 0.264	e	6.64 +/- 0.324	a	**2.94E-09**
	Msr1	8.45 +/- 0.438	b	8.42 +/- 0.351	b	8.20 +/- 0.475	b	8.36 +/- 0.357	b	8.29 +/- 0.381	b	8.20 +/- 0.378	b	8.01 +/- 0.304	b	9.74 +/- 0.150	c	7.25 +/- 0.284	a	**1.08E-06**
	Acta2	3.70 +/- 0.198	ab	3.82 +/- 0.250	ab	3.75 +/- 0.209	ab	3.90 +/- 0.218	b	3.67 +/- 0.285	ab	3.72 +/- 0.322	ab	3.56 +/- 0.217	ab	3.98 +/- 0.216	b	3.37 +/- 0.214	a	**1.97E-02**
2	Fcgr3	8.70 +/- 0.547	bc	8.76 +/- 0.448	c	8.86 +/- 0.521	c	8.83 +/- 0.385	c	8.84 +/- 0.306	c	8.76 +/- 0.234	c	7.71 +/- 0.535	a	7.76 +/- 0.322	ab	7.26 +/- 0.361	a	**5.09E-03**
	Ccr2	6.71 +/- 0.575	bc	6.68 +/- 0.464	bc	7.16 +/- 0.603	c	6.25 +/- 0.501	b	6.32 +/- 0.399	bc	6.54 +/- 0.114	bc	4.13 +/- 0.171	a	4.54 +/- 0.355	a	4.00 +/- 0.166	a	1.26E-01
	Hgf	5.53 +/- 0.700	b	5.42 +/- 0.631	b	5.70 +/- 0.718	b	5.19 +/- 0.454	b	5.26 +/- 0.427	b	5.37 +/- 0.509	b	3.56 +/- 0.148	a	3.97 +/- 0.134	a	3.49 +/- 0.207	a	4.12E-01
	Cd163	4.65 +/- 0.188	b	4.69 +/- 0.295	b	4.81 +/- 0.169	b	4.40 +/- 0.137	b	4.52 +/- 0.298	b	4.61 +/- 0.175	b	3.84 +/- 0.113	a	4.44 +/- 0.153	b	3.82 +/- 0.171	a	**1.94E-06**
	Irf3	5.99 +/- 0.0688	cd	5.97 +/- 0.118	bcd	6.01 +/- 0.157	d	5.70 +/- 0.184	bc	5.68 +/- 0.153	b	5.71 +/- 0.0800	bc	5.24 +/- 0.144	a	5.39 +/- 0.0863	a	5.25 +/- 0.0904	a	5.57E-01
	Mrc1	8.40 +/- 0.304	c	8.34 +/- 0.226	c	8.40 +/- 0.260	c	9.84 +/- 0.139	d	9.83 +/- 0.0867	d	9.76 +/- 0.160	d	1.97 +/- 0.500	a	4.95 +/- 0.537	b	2.20 +/- 0.559	a	**6.68E-09**
	Igf1	6.71 +/- 0.218	c	6.62 +/- 0.174	c	6.83 +/- 0.249	c	7.66 +/- 0.230	d	7.69 +/- 0.345	d	7.93 +/- 0.585	d	3.32 +/- 0.230	a	5.10 +/- 0.202	b	3.26 +/- 0.249	a	**4.63E-07**
	Tlr4	7.28 +/- 0.121	c	7.29 +/- 0.165	c	7.36 +/- 0.134	cd	7.72 +/- 0.125	d	7.70 +/- 0.0737	d	7.71 +/- 0.0988	d	5.99 +/- 0.365	ab	6.34 +/- 0.170	b	5.69 +/- 0.198	a	**1.52E-03**
	Fcgr2b	8.90 +/- 0.494	b	8.94 +/- 0.392	bc	8.93 +/- 0.522	bcd	9.86 +/- 0.385	cdef	9.92 +/- 0.304	df	9.87 +/- 0.322	ef	7.23 +/- 0.701	a	9.18 +/- 0.203	bce	6.72 +/- 0.531	a	**7.43E-10**
	Tlr8	8.16 +/- 0.527	b	8.18 +/- 0.494	b	8.34 +/- 0.558	b	8.32 +/- 0.322	b	8.37 +/- 0.267	b	8.37 +/- 0.239	b	6.84 +/- 0.287	a	7.89 +/- 0.196	b	6.29 +/- 0.212	a	**7.22E-07**
	Cd68	10.0 +/- 0.0648	c	10.1 +/- 0.0818	c	9.98 +/- 0.0934	c	10.1 +/- 0.180	c	10.2 +/- 0.165	c	10.2 +/- 0.171	c	6.97 +/- 0.109	a	8.86 +/- 0.247	b	6.85 +/- 0.343	a	**7.43E-10**
	Fcrls	8.43 +/- 0.716	c	8.07 +/- 0.643	c	8.41 +/- 0.801	c	7.92 +/- 0.664	c	7.97 +/- 0.704	c	7.81 +/- 0.769	c	2.18 +/- 0.376	a	5.87 +/- 0.422	b	2.34 +/- 0.374	a	**2.56E-08**
	Tgfb1	8.44 +/- 0.170	c	8.39 +/- 0.126	c	8.47 +/- 0.248	c	8.39 +/- 0.205	c	8.43 +/- 0.209	c	8.41 +/- 0.216	c	6.73 +/- 0.0799	a	8.02 +/- 0.111	b	6.58 +/- 0.114	a	**1.38E-09**
	Pf4	7.88 +/- 0.136	c	7.77 +/- 0.196	bc	7.85 +/- 0.225	c	7.25 +/- 0.202	bc	7.14 +/- 0.241	b	7.36 +/- 0.439	bc	4.54 +/- 0.436	a	7.43 +/- 0.360	bc	4.61 +/- 0.454	a	**2.48E-09**
	Cntf	3.59 +/- 0.238	ab	3.68 +/- 0.273	ab	3.71 +/- 0.397	ab	3.74 +/- 0.267	b	3.68 +/- 0.367	ab	3.79 +/- 0.217	b	3.20 +/- 0.235	ab	3.16 +/- 0.165	a	3.38 +/- 0.158	ab	9.70E-01
	Ifngr1	8.52 +/- 0.616	c	6.00 +/- 0.630	b	8.70 +/- 0.670	c	8.50 +/- 0.481	c	6.11 +/- 0.621	b	8.53 +/- 0.450	c	6.31 +/- 0.147	b	4.72 +/- 0.196	a	6.22 +/- 0.127	b	**1.17E-02**
	Sbno2	5.92 +/- 0.231	a	5.84 +/- 0.133	a	6.01 +/- 0.286	a	5.86 +/- 0.304	a	5.92 +/- 0.351	a	6.05 +/- 0.356	a	5.87 +/- 0.0656	a	6.02 +/- 0.127	a	5.78 +/- 0.154	a	4.45E-01
	Irf5	7.71 +/- 0.215	ab	7.67 +/- 0.197	ab	7.80 +/- 0.242	ab	7.75 +/- 0.203	ab	7.81 +/- 0.252	ab	7.87 +/- 0.164	ab	7.79 +/- 0.143	ab	7.46 +/- 0.178	a	7.91 +/- 0.152	b	2.74E-01
	Stat6	8.01 +/- 0.119	cd	7.96 +/- 0.0829	bcd	8.02 +/- 0.161	d	7.77 +/- 0.111	ab	7.76 +/- 0.0797	a	7.73 +/- 0.0796	ab	7.73 +/- 0.120	a	7.62 +/- 0.0306	a	7.81 +/- 0.0573	abc	1.57E-01
	Tlr2	6.11 +/- 0.389	b	6.10 +/- 0.434	b	6.21 +/- 0.621	b	4.81 +/- 0.430	a	4.90 +/- 0.412	a	4.60 +/- 0.434	a	4.80 +/- 0.139	a	6.14 +/- 0.579	b	4.62 +/- 0.224	a	**1.20E-04**
3	Ccl24	4.41 +/- 0.925	bc	4.52 +/- 0.916	bc	4.93 +/- 0.863	c	7.09 +/- 0.532	d	7.38 +/- 0.592	d	7.63 +/- 0.394	d	3.13 +/- 0.166	a	3.70 +/- 0.389	abc	3.35 +/- 0.209	ab	5.43E-01
	Pparg	4.80 +/- 0.322	b	4.55 +/- 0.102	b	4.54 +/- 0.181	b	6.00 +/- 0.292	c	6.04 +/- 0.382	c	6.21 +/- 0.401	c	3.30 +/- 0.162	a	3.64 +/- 0.187	a	3.39 +/- 0.195	a	1.81E-01
	Lgals3	5.65 +/- 0.0687	c	5.63 +/- 0.181	c	5.45 +/- 0.113	c	6.95 +/- 0.161	d	6.86 +/- 0.0717	d	6.88 +/- 0.109	d	4.90 +/- 0.375	b	4.33 +/- 0.282	a	4.82 +/- 0.149	b	**1.89E-02**
	Chil3	4.68 +/- 0.570	b	4.64 +/- 0.399	b	4.90 +/- 0.631	b	9.03 +/- 0.317	c	9.20 +/- 0.413	c	9.32 +/- 0.342	c	3.35 +/- 0.153	a	3.63 +/- 0.275	a	3.18 +/- 0.383	a	4.46E-01
	Retnla	1.94 +/- 0.365	a	1.39 +/- 0.458	a	1.72 +/- 0.952	a	11.1 +/- 0.877	b	11.3 +/- 0.983	b	11.5 +/- 0.958	b	1.49 +/- 0.592	a	1.76 +/- 0.294	a	1.43 +/- 0.803	a	7.28E-01
	Irf4	4.50 +/- 0.0716	c	4.38 +/- 0.227	abc	4.37 +/- 0.170	bc	5.77 +/- 0.267	d	5.80 +/- 0.192	d	6.03 +/- 0.196	d	4.00 +/- 0.127	ab	4.49 +/- 0.219	c	3.99 +/- 0.180	a	**2.88E-05**
	Socs2	3.97 +/- 0.721	ab	3.70 +/- 0.661	ab	4.13 +/- 0.780	b	5.65 +/- 0.518	c	5.68 +/- 0.581	c	5.57 +/- 0.509	c	3.39 +/- 0.308	ab	2.91 +/- 0.453	a	3.60 +/- 0.282	ab	4.45E-01
	SiglecF	2.59 +/- 0.329	a	2.52 +/- 0.454	a	2.65 +/- 0.503	a	4.06 +/- 0.679	b	3.96 +/- 0.717	b	4.10 +/- 0.581	b	2.07 +/- 0.184	a	1.91 +/- 0.564	a	1.91 +/- 0.622	a	9.88E-01
	Vim	9.20 +/- 0.162	b	9.19 +/- 0.137	b	9.14 +/- 0.173	b	9.67 +/- 0.155	d	9.61 +/- 0.100	cd	9.72 +/- 0.207	d	9.40 +/- 0.206	bcd	8.70 +/- 0.124	a	9.27 +/- 0.218	bc	**1.76E-03**
	Igf2	1.33 +/- 0.905	a	1.42 +/- 0.996	a	1.51 +/- 0.878	a	3.20 +/- 0.718	bc	3.43 +/- 0.750	bc	3.65 +/- 0.780	c	1.71 +/- 0.568	a	1.53 +/- 0.629	a	1.95 +/- 0.391	ab	9.70E-01
	Il22	1.49 +/- 1.03	a	1.59 +/- 0.390	a	1.74 +/- 0.642	a	3.20 +/- 0.754	bcd	3.47 +/- 0.752	d	3.59 +/- 0.820	cd	2.05 +/- 0.183	ab	2.27 +/- 0.338	abc	2.06 +/- 0.585	ab	9.70E-01
	Col6a1	1.82 +/- 0.286	a	2.09 +/- 0.306	a	1.92 +/- 0.329	a	3.33 +/- 0.668	b	3.84 +/- 0.696	b	3.79 +/- 0.551	b	1.82 +/- 0.215	a	1.94 +/- 0.407	a	2.27 +/- 0.259	a	6.89E-01
	Nfil3	5.66 +/- 0.800	a	5.59 +/- 0.829	ab	5.65 +/- 0.907	ab	7.31 +/- 0.514	c	7.42 +/- 0.528	c	7.28 +/- 0.334	c	6.77 +/- 0.178	bc	4.96 +/- 0.408	a	6.91 +/- 0.187	c	**1.66E-05**
4	Ccr7	1.70 +/- 0.475	ab	1.65 +/- 0.292	ab	1.27 +/- 0.633	a	3.13 +/- 0.724	b	3.04 +/- 1.09	b	3.41 +/- 0.763	b	7.97 +/- 0.498	c	3.28 +/- 1.24	b	8.68 +/- 0.109	c	**3.13E-07**
	Cd40	4.59 +/- 0.219	a	4.57 +/- 0.263	a	4.63 +/- 0.323	a	5.32 +/- 0.121	b	5.43 +/- 0.0904	b	5.30 +/- 0.148	b	8.46 +/- 0.322	d	6.49 +/- 0.313	c	8.82 +/- 0.269	d	**3.48E-09**
	Cxcl9	2.30 +/- 0.172	ab	2.19 +/- 0.418	a	2.33 +/- 0.757	ab	3.09 +/- 0.271	bc	3.31 +/- 0.283	c	2.99 +/- 0.338	abc	9.06 +/- 0.192	e	5.60 +/- 0.342	d	9.13 +/- 0.186	e	**1.38E-09**
	Il6	3.08 +/- 0.150	a	3.25 +/- 0.100	a	2.83 +/- 0.460	a	4.32 +/- 0.421	b	4.22 +/- 0.401	b	4.24 +/- 0.265	b	8.95 +/- 0.416	d	6.46 +/- 0.719	c	9.48 +/- 0.225	d	**2.52E-08**
	Il12b	2.23 +/- 0.493	a	2.66 +/- 0.373	a	2.68 +/- 0.465	a	2.76 +/- 0.486	a	2.88 +/- 0.502	a	2.92 +/- 0.207	a	10.1 +/- 0.890	c	6.63 +/- 0.584	b	11.1 +/- 0.478	c	**4.47E-08**
	Nos2	3.94 +/- 0.113	a	3.87 +/- 0.192	a	3.98 +/- 0.105	a	4.05 +/- 0.393	a	4.21 +/- 0.267	a	3.97 +/- 0.220	a	9.94 +/- 0.129	c	6.34 +/- 0.832	b	10.1 +/- 0.0883	c	**5.00E-10**
	Ly6c1	4.05 +/- 0.400	c	3.97 +/- 0.216	bc	4.34 +/- 0.427	c	3.09 +/- 0.421	a	3.16 +/- 0.282	ab	3.14 +/- 0.357	ab	8.68 +/- 0.304	e	5.58 +/- 0.581	d	8.46 +/- 0.237	e	**3.70E-08**
	Ccl17	2.33 +/- 0.0463	a	2.20 +/- 0.551	a	2.12 +/- 0.398	a	3.32 +/- 0.926	ab	3.06 +/- 1.02	ab	3.05 +/- 0.924	ab	3.15 +/- 0.308	ab	3.81 +/- 0.259	b	3.44 +/- 0.147	ab	6.54E-01
	Ccl22	3.09 +/- 0.458	ab	2.70 +/- 0.378	a	3.00 +/- 0.374	ab	4.66 +/- 1.11	bc	4.51 +/- 1.21	abc	4.09 +/- 1.21	abc	5.83 +/- 0.780	cd	5.01 +/- 0.423	cd	6.63 +/- 0.511	d	1.84E-01
	Socs1	3.28 +/- 0.195	a	3.23 +/- 0.177	a	3.30 +/- 0.363	a	5.14 +/- 0.573	b	5.13 +/- 0.633	b	5.24 +/- 0.771	b	7.32 +/- 0.330	c	5.69 +/- 0.573	b	7.41 +/- 0.482	c	**3.02E-03**
	Lif	2.93 +/- 0.264	ab	2.89 +/- 0.434	a	2.93 +/- 0.325	a	3.67 +/- 0.212	c	3.52 +/- 0.385	bc	3.69 +/- 0.184	c	3.79 +/- 0.154	c	3.68 +/- 0.104	c	3.99 +/- 0.217	c	8.93E-01
	Cd14	7.40 +/- 0.346	b	7.56 +/- 0.472	b	7.55 +/- 0.466	b	6.15 +/- 0.370	a	6.43 +/- 0.515	a	6.32 +/- 0.303	a	9.39 +/- 0.0882	d	8.45 +/- 0.290	c	9.23 +/- 0.113	d	**3.50E-03**
	Cxcl16	7.29 +/- 0.724	ab	7.30 +/- 0.649	ab	7.51 +/- 0.876	bc	6.23 +/- 1.01	ab	6.31 +/- 0.930	ab	6.12 +/- 1.02	a	9.40 +/- 0.0918	d	8.86 +/- 0.0854	cd	9.55 +/- 0.135	d	6.33E-01
	Nfkbiz	4.85 +/- 0.795	ab	4.90 +/- 0.697	ab	5.17 +/- 0.871	b	3.66 +/- 0.466	a	3.79 +/- 0.514	a	3.98 +/- 0.656	ab	6.48 +/- 0.321	c	6.77 +/- 0.219	c	6.72 +/- 0.211	c	9.85E-01
	Ccl3	6.86 +/- 0.560	c	6.83 +/- 0.397	bc	7.01 +/- 0.333	c	5.57 +/- 0.296	a	5.49 +/- 0.323	a	5.88 +/- 0.318	ab	8.31 +/- 0.573	d	9.20 +/- 0.445	e	8.99 +/- 0.229	de	1.43E-01
	Marco	6.15 +/- 0.629	b	5.92 +/- 0.204	b	6.16 +/- 0.315	b	4.85 +/- 0.542	a	4.61 +/- 0.259	a	4.58 +/- 0.357	a	10.1 +/- 0.343	c	10.4 +/- 0.207	c	9.94 +/- 0.290	c	1.45E-01
	Ccl5	3.49 +/- 1.56	a	3.38 +/- 1.45	a	3.92 +/- 1.48	a	2.66 +/- 1.10	a	2.83 +/- 0.928	a	2.67 +/- 1.12	a	12.0 +/- 0.141	b	11.6 +/- 0.183	b	12.1 +/- 0.0454	b	9.50E-01
	Cxcl10	3.60 +/- 0.954	a	3.58 +/- 0.720	a	3.79 +/- 1.58	a	2.85 +/- 0.783	a	3.24 +/- 0.934	a	3.33 +/- 0.603	a	8.96 +/- 0.221	b	8.17 +/- 0.706	b	9.16 +/- 0.230	b	5.89E-01
	Stat1	6.53 +/- 0.293	cde	6.46 +/- 0.279	bde	6.75 +/- 0.417	e	5.97 +/- 0.176	ab	6.01 +/- 0.191	ac	6.00 +/- 0.201	abcd	8.37 +/- 0.136	f	8.48 +/- 0.105	f	8.30 +/- 0.112	f	1.04E-01
	Stat2	5.89 +/- 0.352	a	5.87 +/- 0.291	a	6.12 +/- 0.407	a	5.53 +/- 0.329	a	5.63 +/- 0.372	a	5.72 +/- 0.445	a	7.69 +/- 0.121	b	7.80 +/- 0.244	b	7.81 +/- 0.147	b	9.31E-01
	Il1a	3.40 +/- 0.903	a	3.41 +/- 0.741	a	3.51 +/- 0.838	a	3.27 +/- 0.614	a	3.52 +/- 0.512	a	3.46 +/- 0.421	a	7.72 +/- 0.566	b	8.16 +/- 0.738	b	8.07 +/- 0.632	b	9.31E-01
	Il1b	3.89 +/- 1.74	a	4.30 +/- 1.28	a	4.39 +/- 1.62	a	3.58 +/- 0.943	a	4.05 +/- 1.19	a	3.73 +/- 1.26	a	10.1 +/- 0.264	b	9.48 +/- 0.557	b	10.3 +/- 0.249	b	5.57E-01
	Socs3	3.26 +/- 0.694	a	3.31 +/- 0.533	a	3.73 +/- 0.847	a	3.68 +/- 0.360	a	3.72 +/- 0.418	a	3.79 +/- 0.347	a	6.56 +/- 0.146	b	6.47 +/- 0.161	b	6.50 +/- 0.264	b	8.89E-01
	Il27	2.91 +/- 0.448	a	2.53 +/- 0.585	a	2.98 +/- 0.257	a	2.44 +/- 0.624	a	2.77 +/- 0.348	a	2.74 +/- 0.474	a	5.67 +/- 0.434	bc	4.76 +/- 0.447	b	6.02 +/- 0.378	c	5.18E-02
	Tnf	4.04 +/- 0.473	a	3.93 +/- 0.165	a	4.14 +/- 0.411	a	3.57 +/- 0.253	a	3.86 +/- 0.412	a	3.92 +/- 0.412	a	6.85 +/- 0.682	b	6.90 +/- 0.427	b	7.03 +/- 0.579	b	9.70E-01
	Cxcl11	3.11 +/- 0.252	ab	3.00 +/- 0.417	ab	2.96 +/- 0.337	a	3.96 +/- 0.320	b	3.87 +/- 0.175	ab	3.60 +/- 0.521	ab	7.51 +/- 0.444	c	6.84 +/- 0.832	c	7.75 +/- 0.446	c	1.55E-01
	Il1rn	5.12 +/- 0.167	a	5.19 +/- 0.0485	ab	5.08 +/- 0.396	a	5.90 +/- 0.221	bc	5.97 +/- 0.287	c	5.63 +/- 0.355	abc	8.33 +/- 0.403	d	9.28 +/- 0.487	e	8.39 +/- 0.346	d	**4.54E-02**
	Adgre1	9.60 +/- 0.392	a	9.50 +/- 0.365	a	9.72 +/- 0.428	a	9.61 +/- 0.221	a	9.56 +/- 0.235	a	9.61 +/- 0.274	a	9.89 +/- 0.195	a	10.9 +/- 0.0937	b	9.94 +/- 0.193	a	**5.44E-04**
	Tlr1	5.79 +/- 0.204	a	5.78 +/- 0.276	a	5.92 +/- 0.300	a	5.49 +/- 0.351	a	5.60 +/- 0.135	a	5.55 +/- 0.108	a	6.47 +/- 0.0826	b	7.26 +/- 0.0643	c	6.52 +/- 0.130	b	**1.41E-03**
	Cxcl1	1.90 +/- 0.547	a	2.11 +/- 0.679	a	2.61 +/- 1.00	ab	2.80 +/- 0.581	ab	3.14 +/- 0.763	ab	3.12 +/- 0.735	ab	3.81 +/- 0.435	bc	4.79 +/- 0.638	c	4.67 +/- 0.433	c	7.28E-01
5	Cd86	5.50 +/- 0.756	a	5.41 +/- 0.673	a	5.54 +/- 0.788	a	5.23 +/- 0.774	a	5.37 +/- 0.758	a	5.14 +/- 0.601	a	7.68 +/- 0.335	b	5.69 +/- 0.243	a	7.88 +/- 0.246	b	**3.47E-06**
	Stat3	7.14 +/- 0.376	a	7.11 +/- 0.327	a	7.28 +/- 0.416	a	6.81 +/- 0.264	a	6.87 +/- 0.353	a	6.90 +/- 0.292	a	7.92 +/- 0.115	b	7.13 +/- 0.135	a	8.00 +/- 0.0588	b	**5.40E-04**
	Il10ra	6.46 +/- 0.920	a	6.30 +/- 0.759	a	6.75 +/- 0.955	a	5.89 +/- 0.789	a	5.89 +/- 0.847	a	5.97 +/- 0.946	a	6.88 +/- 0.122	a	6.03 +/- 0.165	a	7.18 +/- 0.128	a	4.86E-01
	Vegfa	6.94 +/- 1.15	abcde	6.94 +/- 0.990	bcde	6.96 +/- 1.12	abcde	6.25 +/- 1.11	abd	6.57 +/- 1.15	bcde	6.12 +/- 0.980	abc	7.74 +/- 0.124	ce	5.06 +/- 0.399	a	8.13 +/- 0.244	de	**1.29E-04**
	Cd80	5.45 +/- 0.476	ab	5.32 +/- 0.508	b	5.52 +/- 0.491	bc	5.55 +/- 0.353	b	5.49 +/- 0.264	bc	5.35 +/- 0.289	ab	6.24 +/- 0.165	cd	4.76 +/- 0.199	a	6.69 +/- 0.278	d	**1.53E-07**
	Tgm2	7.22 +/- 0.497	ab	7.25 +/- 0.459	b	7.40 +/- 0.472	b	7.69 +/- 0.126	bc	7.83 +/- 0.144	bcd	7.80 +/- 0.111	bcd	8.40 +/- 0.513	d	6.50 +/- 0.191	a	8.29 +/- 0.557	cd	**5.68E-07**
	Fgf1	3.17 +/- 0.464	ce	3.15 +/- 0.579	bcde	3.19 +/- 0.592	de	2.35 +/- 0.169	abd	2.19 +/- 0.661	a	2.13 +/- 0.259	abc	3.02 +/- 0.372	abcde	2.77 +/- 0.309	abcde	3.23 +/- 0.225	de	6.89E-01
	Il13ra1	5.56 +/- 0.475	bc	5.54 +/- 0.457	bc	5.76 +/- 0.638	bd	4.28 +/- 0.225	a	4.27 +/- 0.217	a	4.24 +/- 0.146	a	6.55 +/- 0.555	de	5.64 +/- 0.306	bc	6.46 +/- 0.418	ce	**1.78E-02**
	Il4ra	7.97 +/- 0.292	bdef	7.87 +/- 0.376	cde	8.02 +/- 0.421	ef	7.33 +/- 0.235	ac	7.32 +/- 0.256	ab	7.16 +/- 0.205	abcd	8.22 +/- 0.331	ef	8.33 +/- 0.305	f	8.02 +/- 0.184	ef	5.56E-02
	Pdgfb	5.46 +/- 0.225	c	5.38 +/- 0.201	c	5.30 +/- 0.226	bc	4.68 +/- 0.345	a	4.56 +/- 0.321	a	4.51 +/- 0.303	a	5.83 +/- 0.151	c	4.79 +/- 0.235	ab	5.84 +/- 0.241	c	**5.40E-04**
