# Supplementary material for: Temporal changes in macrophage phenotype after peripheral nerve injury
Source: J Neuroinflammation. 2018 Jun 15;15:185. doi: 10.1186/s12974-018-1219-0 (PMC6003127; doi:10.1186/s12974-018-1219-0)
Supplement: Supplementary file 6 — Table S4a. Gene expression of bone marrow-derived macrophages from 3 mouse strains under 3 in vitro stimulation conditions. Log transformed mRNA transcript count mean +/−standard deviation & Tukey letters from linear mixed effect model with mouse as a random effect and an interaction term of strain*stimulation, followed by Benjamini and Hochberg false discovery rate (FDR) correction. p value for the interaction term is displayed. Table S4b. Gene expression of bone marrow-derived macrophages from 2 mouse strains under 3 in vitro stimulation conditions. Log transformed mRNA transcript count mean +/−standard deviation & Tukey letters from linear mixed effect model with mouse as a random effect and an interaction term of strain*stimulation, followed by Benjamini and Hochberg false discovery rate (FDR) correction. (ZIP 128 kb) [file 12974_2018_1219_MOESM6_ESM.zip › Suppl table 4b.pdf]

Supplemental Table 4b: Gene expression of bone marrow derived macrophages from 2 mouse strains under 3 in vitro stimulation conditions. Log transformed mRNA transcript count mean +/-standard deviation & Tukey letters from linear mixed effect model with mouse as a random effect and an interaction term of strain*stimulation, followed by Benjamini and Hochberg false discovery rate (FDR) correction.

Cluster	Stimulation	M(-)				M(IL4)				M(IFNγ+LPS)				
	Strain	BALB/cJ		Il4ra-/-		BALB/cJ		Il4ra-/-		BALB/cJ		Il4ra-/-		FDR
	N	5		5		5		5		5		5		p-value
1	Arg1	3.20 +/- 0.162	a	3.22 +/- 0.291	a	10.8 +/- 0.110	c	3.59 +/- 0.920	a	7.40 +/- 0.202	b	7.09 +/- 0.154	b	**8.92E-11**
	Nfil3	4.84 +/- 0.306	b	4.68 +/- 0.156	ab	6.96 +/- 0.170	c	4.36 +/- 0.195	a	6.68 +/- 0.193	c	6.62 +/- 0.168	c	**2.11E-10**
	Col61a	1.27 +/- 0.512	a	1.56 +/- 0.239	a	3.39 +/- 0.430	b	1.62 +/- 0.396	a	2.78 +/- 0.251	b	2.73 +/- 0.311	b	**1.03E-04**
	Irf5	7.30 +/- 0.173	a	7.18 +/- 0.110	a	7.63 +/- 0.161	b	7.33 +/- 0.0620	a	7.69 +/- 0.106	b	7.59 +/- 0.0352	b	8.55E-02
	Gdnf	1.65 +/- 0.573	a	1.71 +/- 0.522	a	3.08 +/- 0.590	b	1.21 +/- 0.167	a	2.65 +/- 0.269	b	2.59 +/- 0.288	b	**2.24E-05**
	Igf2	1.08 +/- 0.835	a	1.71 +/- 0.313	abc	3.24 +/- 0.655	d	1.12 +/- 0.444	ab	2.09 +/- 0.164	bc	2.22 +/- 0.422	cd	**9.60E-05**
	Il2	1.37 +/- 0.415	ab	1.35 +/- 0.781	ab	3.15 +/- 0.645	c	0.903 +/- 0.630	a	1.63 +/- 0.531	ab	2.48 +/- 0.494	bc	**2.85E-04**
	Il22	1.72 +/- 0.488	ab	1.35 +/- 0.670	a	3.30 +/- 0.386	c	1.25 +/- 0.582	a	2.16 +/- 0.412	ab	2.36 +/- 0.219	bc	**7.30E-04**
	Ccl2	7.42 +/- 0.867	a	8.08 +/- 0.205	abc	8.86 +/- 0.464	c	7.76 +/- 0.723	ab	8.43 +/- 0.177	bc	8.43 +/- 0.154	abc	**2.87E-03**
	Chil3	2.36 +/- 0.259	a	1.55 +/- 0.549	a	8.55 +/- 0.382	b	2.17 +/- 0.673	a	1.84 +/- 0.800	a	2.36 +/- 0.256	a	**3.61E-09**
	Retnla	1.16 +/- 0.714	a	1.67 +/- 0.332	ab	11.2 +/- 0.478	c	2.85 +/- 1.83	b	1.94 +/- 0.362	ab	2.16 +/- 0.337	ab	**4.15E-08**
	Socs2	3.47 +/- 0.434	a	3.46 +/- 0.282	a	5.64 +/- 0.385	b	3.57 +/- 0.277	a	3.77 +/- 0.292	a	4.07 +/- 0.0589	a	**2.45E-06**
	Irf4	4.29 +/- 0.210	bc	4.17 +/- 0.279	abc	5.78 +/- 0.340	d	4.47 +/- 0.162	c	3.87 +/- 0.104	ab	3.80 +/- 0.163	a	**6.80E-05**
	SiglecF	2.21 +/- 0.679	a	2.29 +/- 0.311	a	3.71 +/- 0.298	b	2.09 +/- 0.551	a	2.21 +/- 0.811	a	2.59 +/- 0.191	a	**2.69E-03**
2	Bdnf	1.75 +/- 0.225	a	1.71 +/- 0.452	a	3.13 +/- 0.472	b	1.65 +/- 0.396	a	3.68 +/- 0.414	bc	3.95 +/- 0.205	c	**5.89E-04**
	Ccl22	2.79 +/- 0.537	a	2.74 +/- 0.354	a	4.23 +/- 0.579	b	2.78 +/- 0.421	a	5.83 +/- 0.381	c	6.01 +/- 0.119	c	**1.49E-03**
	Socs1	3.11 +/- 0.371	a	3.11 +/- 0.143	a	5.21 +/- 0.143	b	3.36 +/- 0.295	a	6.97 +/- 0.250	c	6.84 +/- 0.0796	c	**1.42E-06**
	Adgre1	9.00 +/- 0.229	a	8.93 +/- 0.204	a	9.38 +/- 0.0947	b	8.97 +/- 0.142	a	9.55 +/- 0.0431	bc	9.72 +/- 0.0839	c	**2.69E-03**
	Tgm2	6.73 +/- 0.384	a	6.51 +/- 0.247	a	7.21 +/- 0.0570	b	6.81 +/- 0.444	ab	8.60 +/- 0.108	c	8.40 +/- 0.224	c	4.37E-01
	Lif	2.71 +/- 0.378	a	2.89 +/- 0.311	ab	3.20 +/- 0.262	abc	2.67 +/- 0.341	a	3.29 +/- 0.323	bc	3.43 +/- 0.124	c	**2.19E-02**
	Ngf	2.92 +/- 0.191	a	2.93 +/- 0.252	ab	3.34 +/- 0.194	c	2.98 +/- 0.165	ab	3.35 +/- 0.0891	c	3.28 +/- 0.152	bc	1.05E-01
	Il4ra	7.10 +/- 0.292	d	4.93 +/- 0.116	a	6.53 +/- 0.0834	c	5.13 +/- 0.165	a	7.37 +/- 0.130	d	6.08 +/- 0.161	b	**6.80E-05**
	Sbno2	5.25 +/- 0.162	ab	5.20 +/- 0.219	a	5.02 +/- 0.187	a	5.11 +/- 0.153	a	5.57 +/- 0.236	b	5.24 +/- 0.113	ab	9.05E-02
	Ccr7	1.42 +/- 0.184	a	1.27 +/- 0.711	a	2.76 +/- 0.536	b	1.15 +/- 0.346	a	8.57 +/- 0.217	c	8.51 +/- 0.133	c	**2.33E-03**
	Cxcl11	2.75 +/- 0.205	a	2.93 +/- 0.298	a	3.67 +/- 0.233	b	2.97 +/- 0.331	a	8.13 +/- 0.239	c	8.04 +/- 0.0992	c	**6.69E-03**
	Cd40	3.78 +/- 0.132	a	4.20 +/- 0.151	b	4.77 +/- 0.0792	c	4.10 +/- 0.116	b	8.26 +/- 0.190	d	8.50 +/- 0.114	d	**4.46E-07**
	Il6	2.72 +/- 0.211	a	2.92 +/- 0.107	a	3.74 +/- 0.256	b	2.86 +/- 0.127	a	8.55 +/- 0.439	c	8.74 +/- 0.221	c	**5.89E-04**
	Cxcl10	2.44 +/- 0.366	ab	2.22 +/- 0.374	ab	2.86 +/- 0.419	b	1.90 +/- 0.558	a	7.86 +/- 0.323	c	7.59 +/- 0.105	c	9.36E-02
	Il12b	2.49 +/- 0.280	a	2.25 +/- 0.603	a	2.64 +/- 0.831	a	2.19 +/- 0.284	a	9.69 +/- 0.434	b	10.1 +/- 0.227	b	2.26E-01
	Tnf	3.22 +/- 0.227	a	3.05 +/- 0.391	a	3.33 +/- 0.368	a	3.29 +/- 0.343	a	6.96 +/- 0.222	b	7.03 +/- 0.224	b	7.26E-01
	Socs3	2.86 +/- 0.754	a	3.01 +/- 0.321	a	3.73 +/- 0.251	b	3.04 +/- 0.150	ab	6.55 +/- 0.116	c	6.49 +/- 0.0912	c	7.78E-02
	Ccl5	3.06 +/- 0.592	a	2.91 +/- 0.533	a	2.71 +/- 0.346	a	2.69 +/- 0.594	a	11.8 +/- 0.171	b	11.8 +/- 0.139	b	9.36E-01
	Ly6c1	2.25 +/- 0.202	a	2.29 +/- 0.340	a	2.17 +/- 0.459	a	2.06 +/- 0.550	a	8.16 +/- 0.218	b	7.89 +/- 0.243	b	6.62E-01
	Cxcl9	2.29 +/- 0.301	a	2.56 +/- 0.388	a	2.78 +/- 0.248	a	2.30 +/- 0.255	a	8.72 +/- 0.111	b	8.96 +/- 0.0791	b	**1.25E-02**
	Nos2	3.65 +/- 0.229	a	3.86 +/- 0.0915	a	3.89 +/- 0.151	a	3.72 +/- 0.182	a	9.68 +/- 0.0829	b	9.72 +/- 0.0810	b	**2.88E-02**
	Il1a	3.93 +/- 0.196	a	3.93 +/- 0.445	a	3.83 +/- 0.151	a	3.73 +/- 0.250	a	9.10 +/- 0.286	b	9.33 +/- 0.0819	b	3.76E-01
	Il27	2.40 +/- 0.163	a	2.54 +/- 0.262	a	2.34 +/- 0.237	a	2.44 +/- 0.319	a	6.02 +/- 0.225	b	5.89 +/- 0.205	b	3.61E-01
	Marco	3.09 +/- 0.356	a	3.23 +/- 0.224	a	3.20 +/- 0.359	a	3.24 +/- 0.456	a	7.78 +/- 0.219	b	7.59 +/- 0.253	b	3.76E-01
	Il1rn	6.61 +/- 0.207	b	6.66 +/- 0.188	b	6.31 +/- 0.0569	a	6.86 +/- 0.162	b	9.17 +/- 0.0549	c	9.10 +/- 0.121	c	**1.23E-03**
	Stat1	6.17 +/- 0.0754	b	6.21 +/- 0.107	b	5.79 +/- 0.129	a	6.12 +/- 0.101	b	8.06 +/- 0.104	c	7.99 +/- 0.0866	c	**3.63E-04**
	Stat2	6.06 +/- 0.132	b	5.95 +/- 0.0697	b	5.55 +/- 0.184	a	5.83 +/- 0.135	b	7.82 +/- 0.110	c	7.62 +/- 0.137	c	**2.69E-03**
	Stat3	6.72 +/- 0.187	a	6.55 +/- 0.143	a	6.54 +/- 0.151	a	6.59 +/- 0.175	a	7.81 +/- 0.0475	b	7.73 +/- 0.0493	b	1.73E-01
	Cd14	8.15 +/- 0.144	b	8.00 +/- 0.147	b	6.37 +/- 0.175	a	8.04 +/- 0.256	b	9.38 +/- 0.133	c	9.38 +/- 0.0589	c	**4.94E-09**
	Il13ra1	5.24 +/- 0.289	b	5.26 +/- 0.254	b	4.09 +/- 0.109	a	5.28 +/- 0.106	b	6.60 +/- 0.110	c	6.57 +/- 0.0786	c	**2.45E-06**
	Cd86	5.14 +/- 0.385	b	5.07 +/- 0.338	b	4.32 +/- 0.485	a	5.38 +/- 0.414	b	7.42 +/- 0.0968	c	7.38 +/- 0.123	c	**1.16E-04**
	Cxcl16	7.01 +/- 0.369	b	6.83 +/- 0.390	b	6.29 +/- 0.323	a	7.10 +/- 0.185	b	9.35 +/- 0.0655	c	9.55 +/- 0.101	c	**4.32E-03**
	Nfkbiz	4.16 +/- 0.339	b	3.88 +/- 0.374	b	3.21 +/- 0.323	a	4.04 +/- 0.292	b	5.84 +/- 0.206	c	5.86 +/- 0.0524	c	**1.78E-03**
	Il1b	5.95 +/- 0.408	b	5.12 +/- 0.810	b	3.25 +/- 0.418	a	3.88 +/- 0.487	a	11.0 +/- 0.232	c	11.3 +/- 0.0665	c	**5.38E-03**
	Ccl3	6.90 +/- 0.0851	b	6.82 +/- 0.174	b	5.88 +/- 0.165	a	6.81 +/- 0.166	b	8.89 +/- 0.259	c	9.01 +/- 0.121	c	**1.69E-05**
	Cxcl1	3.48 +/- 0.444	a	3.46 +/- 0.340	a	3.23 +/- 0.364	a	3.91 +/- 0.105	a	5.93 +/- 0.625	b	6.39 +/- 0.112	b	1.74E-01
	Cd80	5.35 +/- 0.290	ac	5.22 +/- 0.373	ab	5.29 +/- 0.0612	a	5.27 +/- 0.264	ab	5.74 +/- 0.332	bd	5.89 +/- 0.185	cd	2.52E-01
	Il10ra	5.93 +/- 0.491	bc	5.58 +/- 0.342	ab	5.20 +/- 0.272	a	5.72 +/- 0.490	ab	6.77 +/- 0.112	d	6.57 +/- 0.154	cd	**2.37E-03**
	Tlr1	5.67 +/- 0.180	a	5.62 +/- 0.214	a	5.73 +/- 0.165	a	5.52 +/- 0.132	a	6.10 +/- 0.0508	b	6.09 +/- 0.0692	b	2.32E-01
	Vegfa	5.95 +/- 0.615	a	5.16 +/- 1.02	a	5.31 +/- 0.325	a	5.22 +/- 0.813	a	7.84 +/- 0.216	b	7.89 +/- 0.0984	b	1.43E-01
3	Fcgr3	8.74 +/- 0.358	b	8.61 +/- 0.472	b	8.90 +/- 0.194	b	8.63 +/- 0.299	b	7.32 +/- 0.213	a	7.22 +/- 0.124	a	6.86E-01
	Ifngr1	7.95 +/- 0.387	b	7.62 +/- 0.455	b	8.11 +/- 0.259	b	7.66 +/- 0.348	b	5.73 +/- 0.0982	a	5.79 +/- 0.0631	a	1.02E-01
	Tlr8	8.13 +/- 0.348	bc	7.85 +/- 0.418	b	8.44 +/- 0.161	c	7.79 +/- 0.225	b	6.39 +/- 0.0568	a	6.42 +/- 0.158	a	**4.71E-02**
	Ccr2	5.98 +/- 0.506	c	5.24 +/- 0.336	b	5.85 +/- 0.357	bc	5.43 +/- 0.335	bc	3.53 +/- 0.231	a	3.84 +/- 0.0967	a	**1.57E-02**
	Mrc1	7.76 +/- 0.159	b	7.68 +/- 0.525	b	9.67 +/- 0.0967	c	7.83 +/- 0.313	b	2.72 +/- 0.259	a	2.83 +/- 0.115	a	**9.00E-07**
	Tlr4	7.32 +/- 0.101	b	7.22 +/- 0.187	b	7.76 +/- 0.0516	c	7.29 +/- 0.148	b	5.76 +/- 0.215	a	5.79 +/- 0.140	a	**1.51E-04**
	Fcrls	6.14 +/- 0.336	b	6.05 +/- 0.489	b	6.95 +/- 0.303	c	5.98 +/- 0.548	b	2.43 +/- 0.457	a	2.69 +/- 0.252	a	**3.00E-03**
	Igf1	6.51 +/- 0.348	b	6.28 +/- 0.384	b	7.27 +/- 0.150	c	6.22 +/- 0.110	b	3.79 +/- 0.243	a	3.59 +/- 0.0959	a	**2.47E-03**
	Fcgr2b	8.75 +/- 0.419	b	8.76 +/- 0.474	b	10.1 +/- 0.162	c	8.77 +/- 0.241	b	6.61 +/- 0.247	a	6.65 +/- 0.150	a	**1.61E-04**
	Lgals3	5.45 +/- 0.159	b	5.42 +/- 0.164	b	6.85 +/- 0.0535	c	5.63 +/- 0.102	b	3.92 +/- 0.174	a	3.94 +/- 0.107	a	**4.15E-08**
	Pparg	4.66 +/- 0.0860	b	4.62 +/- 0.225	b	5.86 +/- 0.165	c	4.63 +/- 0.247	b	3.24 +/- 0.260	a	3.19 +/- 0.229	a	**4.46E-07**
	Ccl24	5.73 +/- 0.418	b	4.90 +/- 0.879	b	7.59 +/- 0.474	c	5.12 +/- 0.906	b	3.43 +/- 0.310	a	3.44 +/- 0.172	a	**8.65E-05**
	Cntf	3.34 +/- 0.345	b	3.15 +/- 0.264	ab	3.33 +/- 0.178	b	3.27 +/- 0.173	ab	2.87 +/- 0.120	a	3.12 +/- 0.237	ab	1.31E-01
	Stat6	7.73 +/- 0.161	a	7.70 +/- 0.123	a	7.66 +/- 0.0586	a	7.70 +/- 0.0799	a	7.57 +/- 0.0908	a	7.56 +/- 0.0763	a	7.26E-01
	Cd163	4.36 +/- 0.107	b	4.36 +/- 0.186	b	4.38 +/- 0.231	b	4.57 +/- 0.148	b	3.37 +/- 0.0991	a	3.56 +/- 0.216	a	4.37E-01
	Cd68	10.2 +/- 0.0358	b	10.1 +/- 0.0497	b	10.1 +/- 0.0656	b	10.1 +/- 0.114	b	7.51 +/- 0.297	a	7.48 +/- 0.194	a	9.54E-01
	Tgfb1	8.32 +/- 0.0727	b	8.27 +/- 0.0602	b	8.20 +/- 0.0618	b	8.29 +/- 0.0851	b	6.28 +/- 0.100	a	6.27 +/- 0.0846	a	1.55E-01
	Hgf	5.12 +/- 0.307	c	4.79 +/- 0.400	bc	4.69 +/- 0.206	b	4.90 +/- 0.378	bc	2.98 +/- 0.149	a	3.20 +/- 0.102	a	**2.19E-02**
	Irf3	5.86 +/- 0.117	d	5.83 +/- 0.0865	cd	5.66 +/- 0.127	bc	5.49 +/- 0.0656	b	4.81 +/- 0.129	a	4.85 +/- 0.0800	a	**1.89E-02**
	Pf4	6.22 +/- 0.386	b	6.27 +/- 0.217	b	5.79 +/- 0.152	b	6.11 +/- 0.279	b	4.32 +/- 0.257	a	4.43 +/- 0.429	a	6.56E-01
	Itgam	7.71 +/- 0.141	b	7.69 +/- 0.158	b	7.42 +/- 0.0935	b	7.72 +/- 0.170	b	6.81 +/- 0.261	a	6.58 +/- 0.159	a	**1.06E-02**
	Msr1	8.74 +/- 0.234	bc	9.09 +/- 0.140	c	8.45 +/- 0.138	b	9.09 +/- 0.342	c	7.95 +/- 0.0851	a	7.99 +/- 0.0828	a	**1.06E-02**
	Cd3e	3.31 +/- 0.150	a	2.90 +/- 0.226	a	3.12 +/- 0.465	a	2.93 +/- 0.369	a	2.97 +/- 0.159	a	3.01 +/- 0.209	a	2.44E-01
	Lamc2	3.29 +/- 0.117	a	3.53 +/- 0.131	a	3.17 +/- 0.446	a	3.53 +/- 0.439	a	3.24 +/- 0.160	a	3.18 +/- 0.107	a	2.42E-01
	Ifng	3.12 +/- 0.157	a	3.29 +/- 0.108	a	3.39 +/- 0.221	a	3.31 +/- 0.290	a	3.12 +/- 0.152	a	3.26 +/- 0.109	a	3.61E-01
	Acta2	3.39 +/- 0.258	a	3.69 +/- 0.0635	a	3.62 +/- 0.269	a	3.49 +/- 0.221	a	3.35 +/- 0.165	a	3.40 +/- 0.151	a	8.72E-02
	Il17a	3.27 +/- 0.236	a	3.39 +/- 0.114	a	3.46 +/- 0.222	a	3.42 +/- 0.138	a	3.18 +/- 0.171	a	3.18 +/- 0.193	a	6.86E-01
	Vim	9.50 +/- 0.208	ab	9.43 +/- 0.119	ab	9.72 +/- 0.0683	b	9.45 +/- 0.130	ab	9.40 +/- 0.164	a	9.20 +/- 0.186	a	4.11E-01
	Cxcl13	3.40 +/- 0.220	a	3.68 +/- 0.166	a	3.50 +/- 0.101	a	3.63 +/- 0.277	a	3.50 +/- 0.172	a	3.63 +/- 0.137	a	5.98E-01
	Pdgfb	5.57 +/- 0.109	cd	5.68 +/- 0.210	d	4.83 +/- 0.263	a	5.61 +/- 0.126	cd	5.19 +/- 0.239	ab	5.24 +/- 0.0946	bc	**1.60E-03**
	Tlr2	5.49 +/- 0.225	c	5.44 +/- 0.102	c	3.91 +/- 0.279	a	5.52 +/- 0.126	c	4.45 +/- 0.216	b	4.75 +/- 0.137	b	**9.00E-07**
